# Biological Pathways Leading From ANGPTL8 to Diabetes Mellitus–A Co-expression Network Based Analysis

**DOI:** 10.3389/fphys.2018.01841

**Published:** 2018-12-21

**Authors:** Amnah Siddiqa, Elisa Cirillo, Samar H. K. Tareen, Amjad Ali, Martina Kutmon, Lars M. T. Eijssen, Jamil Ahmad, Chris T. Evelo, Susan L. Coort

**Affiliations:** ^1^Research Centre for Modeling and Simulation, National University of Sciences and Technology, Islamabad, Pakistan; ^2^Department of Bioinformatics - BiGCaT, NUTRIM School of Nutrition and Translational Research in Metabolism, Maastricht University, Maastricht, Netherlands; ^3^Maastricht Centre for Systems Biology(MaCSBio), Maastricht University, Maastricht, Netherlands; ^4^Atta-ur-Rahman School of Applied Biosciences, National University of Sciences and Technology, Islamabad, Pakistan; ^5^Department of Computer Science and Information Technology, University of Malakand, Chakdara, Pakistan

**Keywords:** ANGPTL8, Co-expression network analysis, transcriptomics data analysis, diabetes mellitus, wikiPathways

## Abstract

Angiopoietin like protein 8 (ANGPTL8) is a newly identified hormone with unique nature due to its ability to regulate both glucose and lipid metabolic pathways. It is characterized as an important molecular player of insulin induced nutrient storage and utilization pathway during fasting to re-feeding metabolic transition. Several studies have contributed to increase our knowledge regarding its function and mechanism of action. Moreover, its altered expression levels have been observed in Insulin Resistance, Diabetes Mellitus (Types I & II) and Non Alcohlic Fatty Liver Disease emphasizing its assessment as a drug target. However, there is still a great deal of information that remains to be investigated including its associated biological processes, partner proteins in these processes, its regulators and its association with metabolic pathogenesis. In the current study, the analysis of a transcriptomic data set was performed for functional assessment of ANGPTL8 in liver. Weighted Gene Co-expression Network Analysis coupled with pathway analysis tools was performed to identify genes that are significantly co-expressed with ANGPTL8 in liver and investigate their presence in biological pathways. Gene ontology term enrichment analysis was performed to select the gene ontology classes that over-represent the hepatic ANGPTL8-co-expressed genes. Moreover, the presence of diabetes linked SNPs within the genes set co-expressed with ANGPTL8 was investigated. The co-expressed genes of ANGPTL8 identified in this study (*n* = 460) provides narrowed down list of molecular targets which are either co-regulated with it and/or might be regulation partners at different levels of interaction. These results are coherent with previously demonstrated roles and regulators of ANGPTL8. Specifically, thirteen co-expressed genes (MAPK8, CYP3A4, PIK3R2, PIK3R4,PRKAB2, G6PC, MAP3K11, FLOT1, PIK3C2G, SHC1, SLC16A2, and RAPGEF1) are also present in the literature curated pathway of ANGPTL8 (WP3915^1^). Moreover, the gene-SNP analysis of highly associated biological processes with ANGPTL8 revealed significant genetic signals associated to Diabetes Mellitus and similar phenotypic traits. It provides meaningful insights on the influencing genes involved and co-expressed in these pathways. Findings of this study have implications in functional characterization of ANGPTL8 with emphasis on the identified genes and pathways and their possible involvement in the pathogenesis of Diabetes Mellitus and Insulin Resistance.

## 1. Introduction

Diabetes Mellitus (DM) is a pathological condition which is often characterized by hyperinsulinemia and hyperglycemia and has become a global health challenge for both developed and developing countries (Nanditha et al., 2016). It is estimated to affect 642 million people by 2040 according to the International Diabetes Federation (Zimmet et al., 2016). The underlying pathogenic mechanisms are well studied and encompass deregulated glucose and lipid homeostasis involving inter-organ crosstalk of substrates and hormones (Samuel and Shulman, 2016). However, the suboptimal effectiveness of current diabetic therapies to control pathological glycemic conditions necessitates the identification of novel molecular players involved in regulation of lipid and glucose homeostasis for the development of better pharmacological interventions (Rines et al., 2016). To this end, Angiopoietin like protein 8 (ANGPTL8) is emerging as a novel molecular target for the treatment of DM and related metabolic disorders due to its unique nature in regulating both lipid and glucose metabolism (Siddiqa et al., 2017). Recent studies have demonstrated the upregulation of ANGPTL8 gene expression in various related metabolic disorders including insulin resistance, obesity, DM (type I and II), Metabolic Syndrome, Non Alcoholic Fatty Liver Disease (NAFLD) and Hepatocellular Carcinoma (HCC) emphasizing its assessment as a potential drug target (Chen et al., 2014; Fu et al., 2014b; Hu et al., 2014, 2017; Yamada et al., 2015; Abu-Farha et al., 2016; Guo et al., 2016; Lee et al., 2016; Yin et al., 2017).

ANGPTL8 is a newly identified member of angiopoietin like protein (ANGPTL) family and is also known as lipasin, refeeding induced in fat and liver (RIFL), betatrophin, C19orf80 and TD26 (Ren et al., 2012; Zhang, 2012; Yi et al., 2013). It is induced upon feeding in liver and adipose tissue (both white adipose tissue (WAT) and brown adipose tissue (BAT)) whereas fasting suppresses its expression (Ren et al., 2012; Zhang, 2012; Yi et al., 2013). It has been recognized as one of the essential molecular players involved in the metabolic transition of fasting to re-feeding through both *in vivo* and *in vitro* studies (Ren et al., 2012; Zhang, 2012, 2016). It has been demonstrated to play a role in triglyceride (TG) metabolism by regulating the postprandial lipid traffic via inhibition of lipoprotein lipase (LPL) activity (Ren et al., 2012; Zhang, 2012, 2016; Siddiqa et al., 2016). LPL is a hydrolytic ^1^ enzyme which generates free fatty acids (FFA) from hydrolysis of TGs for subsequent uptake by heart, skeletal muscles and WAT. According to the molecular mechanism demonstrated by Zhang (2016), ANGPTL8 inhibits the postprandial LPL activity of cardiac and skeletal muscles which allows the uptake of FFA by WAT for storage. On the other hand, fasting decreases the expression of ANGPTL8 and in turn the LPL activity in cardiac and skeletal muscles which allows the uptake of FFA by them for energy expenditure. Thus, ANGPTL8 exhibits a significant role in lipid metabolism being a part of lipid partitioning machinery according to nutritional levels. ANGPTL8 has also been demonstrated to play role in other lipid metabolic pathways including adipogenesis and autophagy (Ren et al., 2012; Tseng et al., 2014a). Its role in glucose metabolism was reported in several studies individually (Yi et al., 2013; Fu et al., 2014b; Guo et al., 2016). However, Guo and colleagues demonstrated the mechanism of ANGPTL8 mediated glucose regulation via AKT/GSK3beta and AKT/FOXO arms of insulin signaling pathway (Guo et al., 2016). AKT/GSK3beta and AKT/FOXO signaling regulates the activation of glycogen synthesis and inhibition of gluconeogenesis, respectively.

Recently, we have designed and published an up-to-date literature curated pathway of ANGPTL8 regulation based on its reported regulators and pathways in liver (Siddiqa et al., 2017). The pathway model is available on WikiPathways^1^ (Siddiqa et al., 2017). The pathway allows to clearly visualize the regulatory interactions between different regulators of ANGPTL8 including insulin in presence of glucose, thyroid hormone receptors (THR-alph/beta), sterol regulatory element-binding protein (SREBPs), carbohydrate response element binding protein (ChREBP), mitogen-activated protein kinases (MAPKs), and 5′ AMP-activated protein kinase (AMPK) for its regulation (reviewed in Siddiqa et al., 2017). Moreover, the presence of ANGPTL8 can be visualized in a broader spectrum with respect to other linked pathways including insulin signaling pathway (Ren et al., 2012; Yi et al., 2013; Fu et al., 2014b; Guo et al., 2016), postprandial TG partitioning (Zhang, 2016), adipogenesis (Ren et al., 2012), autophagy (Tseng et al., 2014a), and CD45+ hematopoietic-derived cell proliferation (Cox et al., 2016).

Despite new insights, there is still a great deal of information that remains to be investigated regarding ANGPTL8's functions, regulation and physiological mechanism of action. For example, different studies have indicated the biological processes (such as autophagy, adipogenesis and CD45+ hematopoietic-derived cell proliferation) in which ANGPTL8 is involved but the underlying mechanism of action, associated receptors and the signaling molecules (genes/proteins/metabolites) still remain elusive (Ren et al., 2012; Tseng et al., 2014a; Cox et al., 2016). Besides, the role of ANGPTL8 might not be limited to the already associated biological processes and transcription factors and hence needs further investigation from this point of view as well. Moreover, already identified transcription factors of ANGPTL8 and their coordinated role in initiating its expression during refeeding/fasting metabolic transition also needs further investigation because they have been reported in individual studies.

Briefly, the investigation of predominant functional roles, biological processes, and associated signaling molecules (receptors/cofactors/genes) of ANGPTL8 is of immense importance for its assessment as a molecular target for the treatment of DM and related metabolic disorders. Therefore, the current study was specifically aimed to identify the significantly co-expressed genes with ANGPTL8 and their presence in known pathways (present in WikiPathways) in order to gain mechanistic insights regarding its function. This is because the genes exhibiting similar expression pattern have been demonstrated to be involved in similar functions and/or biological processes besides being co-regulated in previous studies (Oldham et al., 2008; Zhao et al., 2010; Rosen et al., 2011; Konopka et al., 2012). We further explored the co-expressed genes present in a selection of identified pathways to scrutinize their significant Single Nucleotide Polymorphism (SNP) based association with DM and/or other metabolic disorders. The investigation of the effect of SNPs associated with DM can enhance and redefine the gene role in the identified pathways (Cirillo et al., 2017).

Weighted gene co-expression network analysis (WGCNA) is an established method for identification of modules (cluster of genes with similar co-expression patterns) of biologically related genes (Zhang and Horvath, 2005; Langfelder and Horvath, 2008; Zhao et al., 2010). In the present study, we performed WGCNA utilizing a human liver transcriptomics data set retrieved from Gene Expression Omnibus (GEO). The selection of the gene expression data is based on the facts that ANGPTL8 is a predominantly liver expressed gene in humans besides being up-regulated in insulin resistance (Yi et al., 2013; Fu et al., 2014a; Guo et al., 2016), obesity (Fu et al., 2014b) and DM type II (Yamada et al., 2015). Overall, the data set consisted of 21 human liver samples from lean, obese and type II diabetic patients. WGCNA (Zhao et al., 2010) coupled with pathways analysis (Kutmon et al., 2014, 2015; Slenter et al., 2017) as demonstrated in sections below was performed to: (i) identify the genes that are significantly co-expressed with ANGPTL8 in liver, (ii) select Gene Ontology classes that over-represent the hepatic ANGPTL8-co-expressed genes, (iii) identify biological pathways in which the hepatic ANGPTL8-co-expressed genes are present and (iv) investigate whether the DM linked SNPs are present in the ANGPTL8 co-expressed genes. The study focused on the analysis of ANGPTL8 co-expression genes module to increase our knowledge regarding its functions, its pathways based interactions (with co-expressed genes) and its relationship with the other DM related genes. To the best of our current knowledge, this is the first instance to perform a transcriptomics data based analysis for functional assessment of ANGPTL8 in liver.

## 2. Methodology

**Figure 1:**
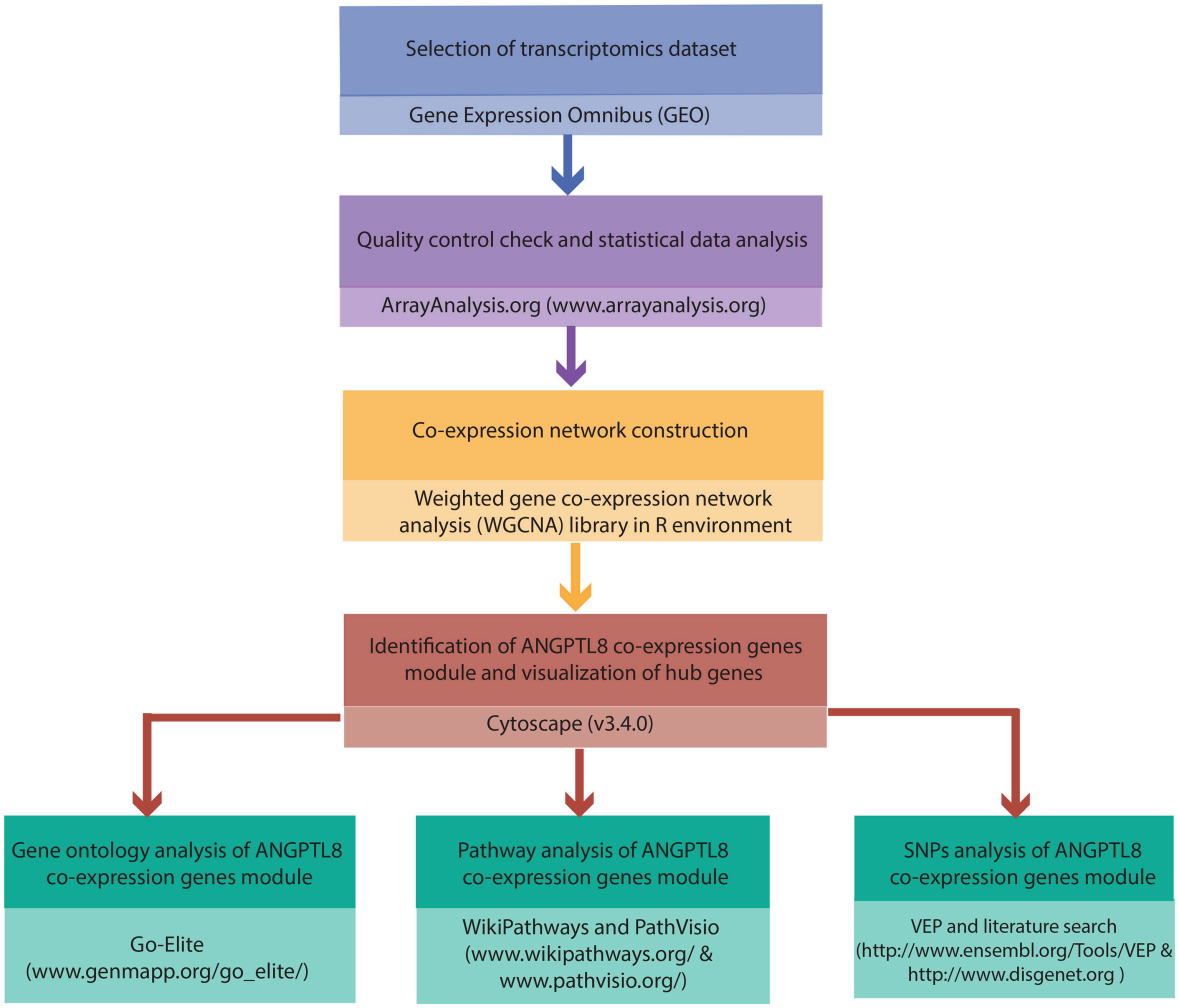
Integrated workflow deployed for functional assessment of ANGPTL8: The steps and the tools/softwares used for the the quality control assessment, construction of ANGPTL8 co-expression network, Gene Ontology analysis, pathway analysis and variant effect prediction analysis (through SNPs identification) are described.

The complete work flow employed in the current study is illustrated in Figure 1.

### 2.1. Selection of Transcriptomics Data Set

Liver is the predominant expression site of ANGPTL8 that is also over-expressed during insulin resistance (Yi et al., 2013; Fu et al., 2014a; Guo et al., 2016), obesity (Fu et al., 2014b) and DM type II (Yamada et al., 2015). Therefore, the selection of a data set in which all of these conditions are present could aid in the identification of highly correlated genes with ANGPTL8 based on similar expression pattern observed across all the samples. A systematic and thorough check of GEO database (Barrett et al., 2012) was performed for the selection of a suitable data set as described above. The gene expression profiles of human liver samples with GEO ID: GSE64998 was selected out of the identified data sets (GSE15653, GSE23343, and GSE64998) based on the best quality and appropriate sample size for performing co-expression network analysis. It consists of six healthy control samples, eight obese non-diabetic and seven type 2 diabetic patient samples and was performed in GPL11532 (Affymetrix Human Gene 1.1 ST Array Platform). This data set had been already analyzed with a different approach and aim than ours by Kirchner et al. (2016). Several clinical parameters associated with the samples are also provided comprehensively by Kirchner et al. (2016).

### 2.2. Quality Control Check and Statistical Data Analysis

The raw data of GSE64998 was downloaded and reanalyzed using ArrayAnalysis.org (Eijssen et al., 2013). ArrayAnalysis.org is a web server to perform quality control, preprocessing and statistical analysis of microarray data. We selected Entrez IDs for gene annotation of microarray probe IDs via ArrayAnalysis.org. The quality control and preprocessing report obtained is provided as Supplementary Presentation 1. The data was normalized using Robust Multi-array Average (RMA) method and is provided as Data Sheet 1. All the samples of GSE64998 were included for the subsequent analysis as there were no outliers. Average expression of less than 5 was selected as cutoff value to remove the genes with low expression values from the data set which resulted in selection of 10869 genes (Data Sheet 2).

### 2.3. ANGPTL8 Co-expression Network Construction

The weighted gene co-expression network analysis (WGCNA) is an established systems biology method for construction of correlation networks based on similar gene expression patterns observed across microarray samples (Zhang and Horvath, 2005). It allows the identification of co-expression genes modules (set of genes observed with similar correlation pattern) from gene expression data through unsupervised learning methods. The method was implemented using the R package “WGCNA” (Langfelder and Horvath, 2008) in order to identify the ANGPTL8 co-expression genes module. The preprocessed normalized data of all samples (Data Sheet 2) obtained in previous step was used as an input. We selected automatic network construction and module detection method to perform WGCNA (Langfelder and Horvath, 2008). The complete R code utilized to perform the analysis is provided in Data Sheet 3.

As a first step, a similarity matrix was constructed by measuring Pearson's correlation for all gene pairs. Next, an adjacency matrix was constructed by raising the similarity matrix to the soft thresholding power beta (Equation 1) Zhao et al. (2010).

(1) $$a(i,j)=\;|cor(x(i),x(j){|}^{\beta }$$

where *x(i)* and *x(j)* corresponds to expression values of gene *i* and gene *j*, respectively. The soft thresholding power beta is selected in order to achieve the approximate scale-free network topology as described in Langfelder and Horvath (2008). We selected power of *beta* = 14 to fulfill the scale free topology criterion. This adjacency matrix was converted into a Topological Overlap Measure (TOM) matrix where TOM is a highly robust network proximity measure (Zhang and Horvath, 2005; Langfelder and Horvath, 2008) (Equation 2). Next, TOM matrix was converted into a dissimilarity TOM matrix (Equation 3) which was subsequently used to create a dendrogram through average hierarchical clustering method. Lastly, the dynamic branch cutting algorithm was applied on the dendrogram in order to obtain the clusters (modules) of highly correlated genes.

(2) $$\begin{array}{r} TOM_{ij}=\frac{\sum_{u}a_{iu}a_{uj}\;+\;a_{ij}}{min\left(k_{i},k_{j}\right)\;+\;1\;-\;a_{ij}} \end{array}$$

where *a*_*iu*_, *a*_*uj*_ and *a*_*ij*_ represents the adjacency function based values between gene pairs *(i,u) (u,j)* and *(i,j)*. *k*_*i*_, *k*_*j*_ represents the connectivity of genes *i* and *j*, respectively.

(3) $$\begin{array}{r} DistTOM_{ij}=1-TOM_{ij} \end{array}$$

The co-expression genes module identified with the presence of ANGPTL8 was selected for further analysis and it was exported in Cytoscape (Shannon et al., 2003) network format using the WGCNA R function “exportNetworkToCytoscape.” This function allows to remove the edges with lower TOM values based on the value of the parameter named “threshold.” We used a threshold value equal to 0.02 for removing the low weighted edges from the ANGPTL8 genes co-expression module. The module-trait relationship was not assessed because we were not interested to relate the modules with a single phenotype as already described in section 2.1.

### 2.4. Identification of Hub Genes of Co-expression Genes Module of ANGPTL8

Next, the co-expression genes module of ANGPTL8 (identified in the previous step) was visualized as a network using the network visualization and analysis software Cytoscape (version 3.4.0) (Shannon et al., 2003).

For the identification of hub genes in the ANGPTL8 related co-expression genes module, we used the connectivity (degree centrality) as described by Langfelder and Horvath (2008). In an undirected network, the degree centrality of a node (gene/protein/metabolite etc) can be defined as the total number of the edges incident on the node. Genes of ANGPTL8 co-expression module with a degree greater than or equal to 80^th^ percentile were considered as the hub genes (Barabási et al., 2011). The hub genes are the most representative genes in a co-expression network due to the maximum number of co-expressed genes linked with them (Langfelder and Horvath, 2008).

### 2.5. Gene Ontology (GO) Analysis of Co-expression Genes Module of ANGPTL8

Gene Ontology (GO) analysis aids in inferring the gene properties from the controlled vocabulary (defined terms) maintained by GO project (Botstein et al., 2000). Every gene product is classified based on three types of ontologies i.e., biological process (BP), molecular function (MF) and cellular compartment (CC). We used GO-Elite (Team, 2017b) version 1.2.5 to perform GO analysis (Zambon et al., 2012). GO-Elite is a software which identifies minimal non-redundant set of GO terms describing a given set of genes. We compared the genes present in the genes module identified with ANGPTL8 with all the measured genes. We used the following settings for GO analysis: (i) 2000 permutations, (ii) Z-score threshold > 1.96, (iii) *p*-value threshold < 0.05 and (iv) minimum number of changed genes is three. We used Cytoscape (version 3.4.0) (Shannon et al., 2003) for intuitive visualization of the results in order to analyze the connections between the genes and identified GO terms (Figure S1).

### 2.6. Pathway Analysis of Co-expression Genes Module of ANGPTL8

We investigated the presence of genes identified within the ANGPTL8 co-expression network in the complete curated human pathway collection (*n* = 710) of WikiPathways (Slenter et al., 2017). All pathways were scrutinized for the presence of at least one of the ANGPTL8 co-expression module genes. PathVisio, was used for the visualization of the selected pathways (Kutmon et al., 2015). The pathway analysis was performed in order to allow us to (i) determine the biological processes that might be the part of physiological mechanisms associated with ANGPTL8 and the significant genes co-expressed with it; (ii) determine the unknown genes/proteins co-expressed with ANGPTL8 from biological processes already known or associated with it.

### 2.7. Single Nucleotide Polymorphism (SNP) Analysis of Selected Co-expressed Genes With ANGPTL8

We identified SNPs associated with DM and other metabolic disorders that are present in 72 ANGPTL8 co-expressed genes in a selection of 10 pathways found with the highest number of co-expressed genes. The analysis was performed using DisGeNET database (Team, 2017a) version 4.0. The names of 72 genes were provided as input in the gene search panel of the DisGeNET website, in which the top 10 disease-association list and the top 10 disease-associated variants list for each gene, were further consulted. We extracted the names and IDs of the diseases associated to the genes that reported the highest DisGeNET score (Data Sheet 4). However, if in the top 10 disease-associations, a disease related to DM and other metabolic disorders was listed with a lower score, its name, ID and score was also included in Data sheet 4. Moreover, in this table SNPs associated to DM and other metabolic disorders are also reported for several genes, with the name and ID of the associated disease and the DisGeNET score related to the strength of the association. The DisGeNET score ranges from 0 to 1 and it ranks the gene-disease associations taking into account the number and type of sources (level of curation, organism), and the number of publications supporting the association. The effect of the variants in the genes and the pathways were further investigated with literature search in Google and consultation of several databases such as: Ensembl (Fernández and Birney, 2010) and SNPedia (Team, 2017c).

## 3. Results

### 3.1. Identification of ANGPTL8 Co-expression Genes Module and Visualization of Hub Genes

**Figure 2:**
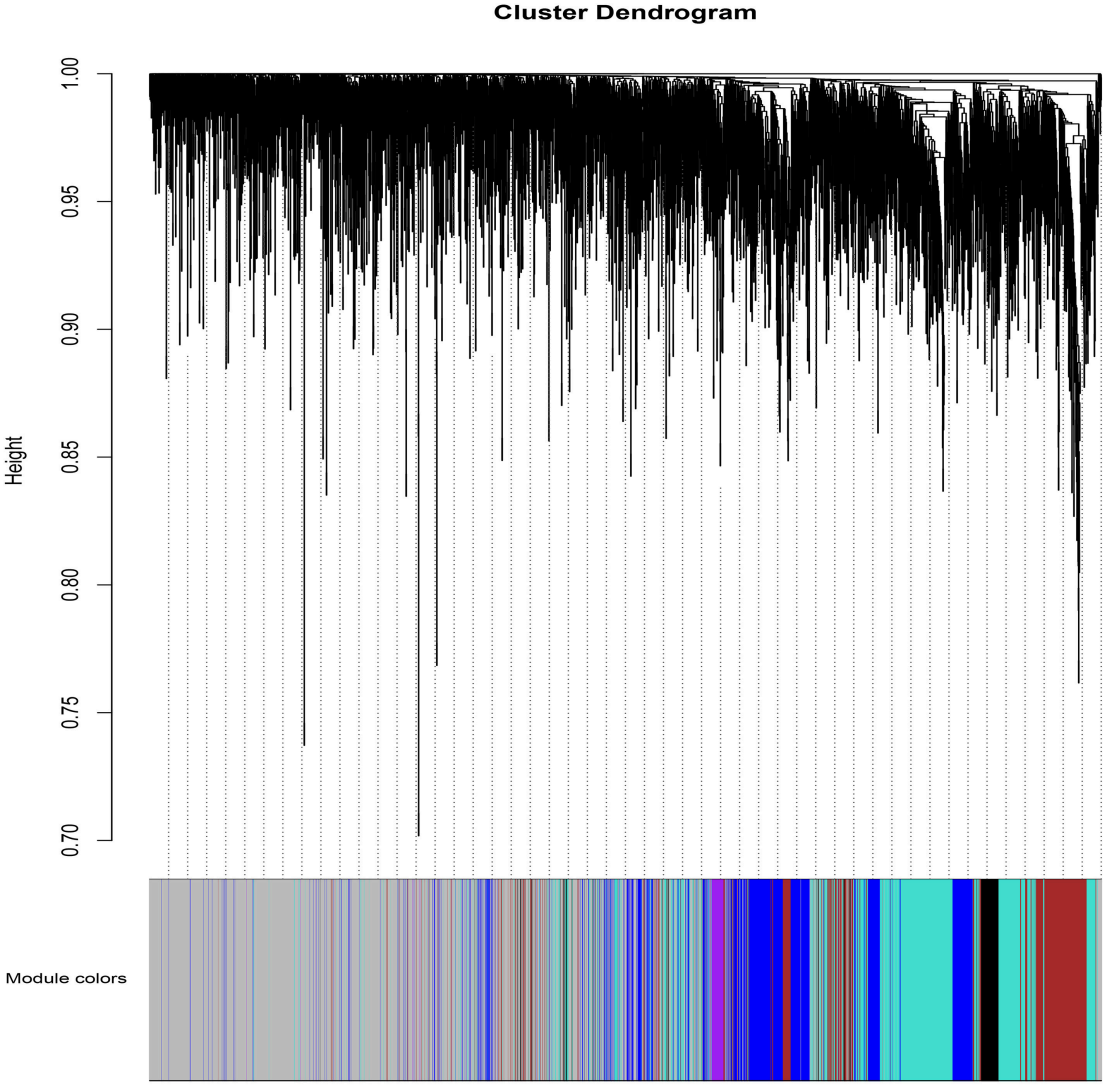
The dendrogram of dissimilarity TOM Matrix constructed using hierarchical clustering. Each vertical line corresponds to the gene. Module colors are provided at the bottom. Each color corresponds to the separate genes module.

WGCNA (Zhang and Horvath, 2005; Langfelder and Horvath, 2008; Zhao et al., 2010) was applied to gain insights into the functional organization of ANGPTL8 and its associated co-expressed genes in human liver utilizing a transcriptomics data set of lean, obese and DM type II subjects (available online at^2^) (Kirchner et al., 2016). ANGPTL8 is a predominantly liver expressed gene in humans which has been found up-regulated in insulin resistance (Yi et al., 2013; Fu et al., 2014a; Guo et al., 2016), obesity (Fu et al., 2014b) and DM type II (Yamada et al., 2015). Therefore, a data set expressing all these conditions was selected in order to allow the selection of highly correlated genes with ANGPTL8 across all the samples and conditions. The expression profile of 10869 unique genes (Data Sheet 2) obtained after normalization and filtering off the probes with low intensities were used to construct the gene co-expression network by applying the steps described in the section 2. Twelve gene modules (clusters of highly co-expressed genes) other than gray module (unclustered genes) were obtained by applying automatic module detection and dynamic tree cutting algorithm with minimum cluster size of 30. The graphical illustration of the resultant dendrogram, obtained from the hierarchical clustering based on the dissimilarity TOM matrix, is given in Figure 2. The number of genes in the corresponding modules with the respective color codes are provided in Table 1. The complete list of the genes (with respective Entrez ids) identified in each module is provided in Data Sheet 5.

**Table 1:** Modules resulted from the hierarchical clustering: Module names are assigned colors and module size corresponds to the number of genes clustered in each module.

**Module name**	**Module Size**	**Module name**	**Module Size**
Turquoise	999	Black	371
Blue	783	Pink	314
Brown	600	Magenta	183
Yellow	498	Purple	76
Green	462	Greenyellow	64
Red	461	Tan	43

The red module was identified with ANGPTL8 and 460 other genes and was then exported in cytoscape (Shannon et al., 2003) network format for subsequent visualization and analysis. This network is composed of 447 nodes and 1781 interactions due to the filtering criteria used for removing the edges with the lower TOM values. It will be referred as co-expression network of ANGPTL8 from here onwards. The graphical illustration of the entire co-expression network is provided as Figure S2. The topological analysis of this network revealed that 97 genes were greater than or equal to 80th percentile according to the degree (Figure S2). These genes are classified as hub genes and represent the most highly connected nodes in the entire network. The hub genes in a co-expression network are important to gain insights into the associated functional roles (phenotypic outcomes) related to majority of the genes. It is because these genes show highly similar co-expression patterns and often are part of similar biological functions, biological process and/or are co-regulated (Langfelder and Horvath, 2008).

Ubiquitin protein ligase E3 component n-recognin 2 (UBR2) is the gene with the highest degree in the entire co-expression network of ANGPTL8. It is a part of the N-end rule pathway which regulates proteolysis of intracellular proteins on the basis of identity of their N-terminal amino acids (Gibbs et al., 2014). This pathway is found conserved from yeast to eukaryotes and is important determinant of half-life of diverse set of proteins. It has been previously demonstrated to serve various developmental and physiological processes including fidelity of chromosome segregation, apoptosis, autophagy, cardiovascular development in animals, regulation of cellular check point controls (by degradation of regulatory proteins involved in cellular differentiation, division and programmed cell death) quality control of cytosolic proteins, controlling the redox dynamics of stress related cellular compounds (such as nitric oxide, thiols, heme, oxygen, and others) and leaf senescence in plants (reviewed in Lee et al., 2015). Additionally, it has been demonstrated to play an inhibitory role in mTOR signaling pathway (Kume et al., 2010). It is interesting to observe that ANGPTL8 is a part of insulin and glucose mediated signaling pathway which also includes downstream regulators of mTOR signaling arm (Siddiqa et al., 2017). Moreover, one of the outstanding questions that remained elusive regarding ANGPTL8 was the identity of its degradation pathway pointed out by Zhang (2016). The results of current analysis demonstrate the possible involvement of N-end rule pathway (through UBR2) in degradation of ANGPTL8 which should be further investigated through wet-lab studies.

Other top nine hub genes based on the degree are KANSL1L, ORC2, AGL, BNIP2, MET, MBTD1, TFPI, ALDH6A1, and SLC16A4 (Figure S2). The role of KANS1L, ORC2, MBTD1, BNIP2, MET, TFPI is mainly associated with DNA replication and/or cellular division; AGL and ALDH6A1 are enzymes involved in metabolic pathways and SLC16A4 is a solute transporter protein (Naldini et al., 1991; Bao et al., 1996; Carpenter et al., 1996; Luo et al., 2013; Norling et al., 2014; Espada et al., 2017).

ANGPTL8 itself is connected with nine other genes in its co-expression network which means they are the most strongly co-expressed genes with it (Table 2). Two of these genes are also identified as hub genes i.e., tissue factor pathway inhibitor (TFPI) and insulin-like growth factor-binding protein 1 (IGFBP1). TFPI plays an important role in the regulation of blood coagulation pathway (Dong et al., 2011). It is an inhibitor of tissue factor (TF) which is a glycoprotein present on surface of macrophages and other extravascular cells. TF is involved in positive induction of inflammatory cytokines (such as TNFα, IL-1 and IL-6) and coagulation signaling cascade. Thus, TFPI plays a protective role in maintaining cellular and systemic homeostasis of immune system. Additionally, TFPI has been demonstrated to be involved in three interdependent biological processes that is coagulation, angiogenesis and lipid metabolism (Holroyd and Simari, 2010). Excess cellular lipid forms lipotoxic metabolites (such as cholesterol crystals) which on one hand induce inflammatory cytokine production and on the other induce TFPI (Holroyd and Simari, 2010; Espada et al., 2017). TFPI not only regulates the inflammatory processes through their inhibition but also reduces cholesterol concentration (through stimulation of internalization and degradation of VLDLs through HSPG-dependent pathway) (Holroyd and Simari, 2010). ANGPTL8 has also been previously demonstrated as an integral component of lipid metabolism. Therefore, these results imply that TFPI and ANGPTL8 represent interesting multifunctional molecular players which might be mutually involved in maintaining the interconnected physiological feedback mechanisms between angiogenesis, coagulation and lipid metabolism. These feedbacks should be investigated further to understand the role of these genes in the integrated physiological pathways for maintaining homeostasis.

**Table 2:** Neighbors of ANGPTL8 in its co-expression network.

**Gene Symbol**	**Full Gene Name**	**Degree**
TFPI	Tissue factor pathway inhibitor	42
IGFBP1	Insulin like growth factor	
	binding protein 1	21
YKT6	YKT6 v-SNARE homolog	10
PPARGC1A	PPARG coactivator 1 alpha	6
MID1IP1	MID1 interacting protein 1	4
C10orf10	chromosome 10 open reading frame 10	3
BHLHE40	basic helix-loop-helix family member e40	3
VPS18	VPS18, CORVET/HOPS core subunit	3
SDF2L1	stromal cell derived factor 2 like 1	2

*Degree represents the number of connected nodes (genes) with respective genes in the ANGPTL8 co-expression network*.

IGFBP-1 is a plasma carrier protein which binds to insulin-like growth factors (IGFs) I and II and increases their half-life (Firth and Baxter, 2002; Forbes et al., 2012). IGFI and IGFII are ligands of IGF signaling system involved in cell proliferation, differentiation, migration and metabolic processes. These ligands (IGF I and II) can bind with IGF-I and II receptors, isoforms of insulin-receptors and their hybrid receptors (Belfiore et al., 2009). IGFBP-1 has also been demonstrated to improve whole body glucose regulation through its role in integrin mediated signaling cascade (Haywood et al., 2017). IGFBP1 can bind to integrins (transmembrane cellular adhesion proteins/receptors) through its Arg-Gly-Asp (RGD) domain and activate focal adhesion kinase (FAK) (Lebrun et al., 1998; Haywood et al., 2017). FAK signaling converges with insulin/insulin like growth factors signaling at IRS-1 phosphorylation signaling point. Previous studies have shown that IGFBP-1 induced improved glucose regulation and increased insulin sensitivity is a part of protective mechanism induced upon insulin resistance in the body (Haywood et al., 2017). These results are crucial for further investigation with reference to the similar role of ANGPTL8 demonstrated for improved glucose tolerance in insulin resistance condition by Guo et al. (2016). Their data indicated that ANGPTL8 is increased in the presence of both glucose and insulin which subsequently induces the phosphorylation of AKT involved in improving glucose tolerance through inhibition of gluconeogenesis (via phosphorylation of FOXO) and induction of glycogen synthesis (via phosphorylation of GSK3beta). The signaling events leading to induction of ANGPTL8 were verified in several other studies as well and can be visualized in the recently curated ANGPTL8 regulatory pathway present in WikiPathways (Siddiqa et al., 2017). However, the mechanism of action of phosphorylation induced activation of AKT via ANGPTL8 being direct or indirect (involving other genes/proteins than ANGPTL8) is still a quest. Therefore, it would be interesting to investigate the connection between IGFBP-1 and ANGPTL8 in improving glucose tolerance in insulin resistance since both are higly co-expressed with each other and are part of overlapping signaling pathways (focal adhesion pathway and insulin/IGF signaling pathway).

Among other neighbors, peroxisome proliferator-activated receptor gamma coactivator 1-alpha (PPARG- C1A) is a key regulator of mitochondrial biogenesis. It integrates vast set of physiological stimuli (including growth factors, stress, cold exposure, cytokines, exercise etc.) into respective metabolic responses involving fat and glucose metabolism (Jornayvaz and Shulman, 2010). It is a key co-activator of several transcription factors including NRFs, peroxisome proliferator-activated receptor (PPAR), thyroid hormone, glucocorticoid, estrogen and estrogen-related receptors (ERRs) alpha and gamma (Ventura-Clapier et al., 2008). This result is in line with previously demonstrated regulators of ANGPTL8 including thyroid hormone receptor alpha and beta, Liver X receptor (LXR) and PPAR (reveiewd in Siddiqa et al., 2017). Synaptobrevin homolog YKT6 (YKT6) and vacuolar protein sorting-associated protein 18 homolog (VPS18) are two other neighbors of ANGPTL8 in the co-expression network which are involved in vesicular transport of cytoplasmic proteins within different cellular locations. VPS18 is specifically involved in the vesicles transport of endosome/lysome pathway (Huizing et al., 2001). ANGPTL8 itself is a secreted protein which was demonstrated to reside in lysosomal vesicles like compartments in cytoplasm and these proteins (YKT6 and VPS18) might represent its associated partner molecules during vesicular transport process (Tseng et al., 2014a). Among other neighbors, MID1 Interacting Protein 1 (MID1IP1) is involved in hepatic lipogenesis (Tsatsos et al., 2008) and microtubule stabilization during cell division (Berti et al., 2004), stromal cell-derived factors 1-alpha and 1-beta (SDF2L1) is a chemokine protein playing role in hematopoiesis (Bleul et al., 1996; Ara et al., 2003), Class E basic helix-loop-helix protein 40 (BHLHB2) plays role in cell differentiation and control of circadian rythm, and chromosome 10 open reading frame 10 (C10orf10) plays role in regulation of autophagy (Salcher et al., 2017). These results (identified co-expressed genes) are in line with the roles associated with ANGPTL8 including lipid metabolism (Zhang, 2012, 2016), hematopoiesis (Cox et al., 2016), autophagy (Tseng et al., 2014a), and circadian rhythm (Dang et al., 2016). Overall, the results of the co-expression network analysis revealed the genes with similar roles observed for ANGPTL8 in previous studies. Therefore, these genes represent a focused and tremendous knowledge body for further investigations regarding functional insights of ANGPTL8.

### 3.2. Gene Ontology Analysis

Gene Ontology (GO) analysis was performed to find the significant GO terms associated with the genes present in co-expression genes module of ANGPTL8 using GO-Elite (Zambon et al., 2012). Thirty Two biological processes, seven molecular function and six cellular components GO terms were identified to be associated with co-expression genes module of ANGPTL8 (Data Sheet 6). The graphical illustration of the significant GO terms along with the associated genes is provided as Figure S1. Overall, the significant GO terms of biological processes were identified related to different metabolic processes. Top five biological processes on the basis of degree (number of connections of a node) include carbohydrate metabolic process (GO:0005975), monocarboxylic acid metabolic process (GO:0032787), regulation of small GTPase mediated signal transduction (GO:0051056), lipid modification (GO:0030258) and phosphatidylinositol biosynthetic process (GO:0006661). ANGPTL8 is found associated with carbohydrate metabolic process (GO:0005975), that is the largest connected metabolic process in the entire network (Figure S1). Previous studies have demonstrated the role of ANGPTL8 in different metabolic processes including carbohydrate and lipid metabolism (reviewed in Zhang and Abou-Samra, 2013; Tseng et al., 2014b; Siddiqa et al., 2017). Other than the metabolic processes, several other biological processes were also identified including leukocyte migration (GO:0050900), extracellular matrix disassembly (GO:0022617), blood vessel development (GO:0001568), regulation of epithelial cell migration (GO:0010632) and regulation of epithelial to mesenchymal transition (GO:0010717). These biological processes are especially relevant to a recently revealed role of ANGPTL8 in stimulation and proliferation of CD45+ hematopoietic derived cells demonstrated by Cox et al. (2016). CD45 is a glycoprotein also known as receptor-type tyrosine-protein phosphatase C (PTPRC) which is present at the surface of leukocytes and their progenitor hematopoietic stem cells (Trowbridge and Thomas, 1994). It plays important role in different hematopoiesis related processes including cellular differentiation, migration and proliferation of hematopoietic stem cells (HSCs). It is a key signaling component of B- and T-cell activation. Since the underlying signaling pathways and genes of ANGPTL8's role in proliferation of CD45+ derived cells remained elusive, the co-expressed genes of ANGPTL8 identified in these biological processes (provided in Data Sheet 6) represent peculiar molecular targets for future investigation. The associated cellular compartment related GO terms includes mitochondrial matrix , (GO:0005759), microtubule (GO:0005874), actomyosin (GO:0042641), cell-cell junction (GO:0005911), cytoskeleton (GO:0005856) and microtubule organizing center part (GO:0044450). Mitochondrial matrix is a cellular site involving fatty acid oxidation and other energy expenditure related processes whereas the other identified compartments (microtubule, actomyosin, cytoskeleton, microtubule organizing center part) are involved in the cellular motility, maintaining cellular shape and cell division. These results are in line with the biological processes identified with the genes present in the co-expression module of ANGPTL8 as discussed above. Finally, the main molecular functions identified in ANGPTL8 co-expression network are phospholipase activity (GO:0004620), monocarboxylic acid transmembrane transporter activity (GO:0008028), ion channel binding (GO:0044325), protein binding, bridging (GO:0030674), nucleoside-triphosphatase regulator activity (GO:0060589), protein homodimerization activity (GO:0042803), and transferase activity (GO:0016740).

### 3.3. Pathways Analysis

The genes in the ANGPTL8 co-expression network were further investigated for their presence in the complete curated WikiPathways collection. WikiPathways is a public repository of curated and dynamic models of biological processes (Slenter et al., 2017). A total of 474 human pathways were identified to contain at least one of the genes from the co-expression network of ANGPTL8. Whereas, 258 genes from co-expression network of ANGPTL8 were identified to be present and 189 genes were identified to be not present in any of these identified pathways. The complete list of pathways along with the respective genes found in them is provided as Data Sheet 7 and entire gene to pathway network of these results is graphically illustrated in (Figure S3).

**Figure 3:**
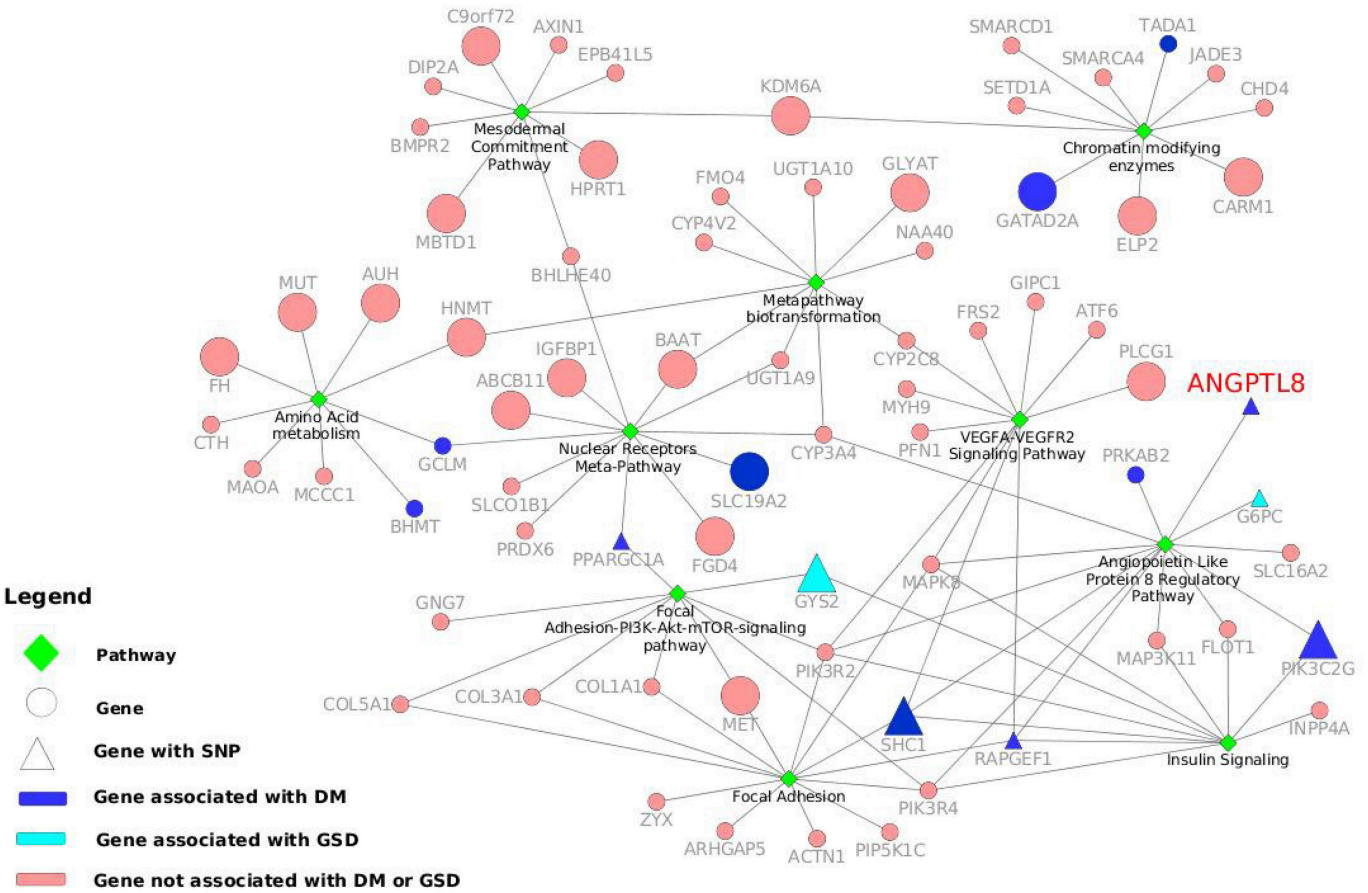
Gene-pathway network with information of genes and variants associated to DM and other metabolic disorders. The top ten pathways identified with maximum number of genes in WikiPathways human curated collection are illustrated as green diamonds. The pathways are connected with seventy two genes (circles and triangles) and the size of the gene nodes indicate that the gene is either a hub gene (large nodes) or not (small nodes) in the co-expression network of ANGPTL8. The dark blue nodes indicate genes associated with DM. The aqua color nodes are the genes associated with GSD1. The triangle shapes represent the genes with a SNPs associated to DM or other metabolic disorders. ANGPTL8 gene is highlighted in red.

Ten of these pathways identified with maximum (above 9) number of genes are listed in Table 3 and represent highly associated biological processes with ANGPTL8. The gene-pathway network of these 10 pathways (subset derived from the complete gene to pathway network in Figure S3) is graphically illustrated in Figure 3. The network is composed of a total of 72 genes and 10 pathways connected with shared genes among them. Twenty two hub genes identified in this network are shown with large size as compared to non-hub genes in the network. Two pathways including the angiopoetin like protein 8 regulatory pathway (WikiPathways ID: WP3915) and Focal Adhesion pathway (WikiPathways ID: WP306) were identified with the presence of thirteen genes (maximum number of genes per pathway in this analysis) each. Both of these pathways share several genes among them including PIK3R2, PIK3R4, MAPK8, SHC1, RAPGEF1. Previous studies have demonstrated the role of both of these pathways in improving glucose tolerance and insulin sensitivity in insulin resistance condition (Lebrun et al., 1998; Guo et al., 2016; Haywood et al., 2017). Besides, IGFBPI has been identified as a highly co-expressed gene with ANGPTL8 (as mentioned in sections above) which induces focal adhesion pathway through its RGD domain (Lebrun et al., 1998; Haywood et al., 2017). Therefore, further studies are required to investigate the interdependence of ANGPTL8 signaling pathway and focal adhesion signaling pathway in regulating glucose homeostasis especially in pathological conditions like insulin resistance and DM. These results emphasizes the association of revealed molecular players with ANGPTL8 which should be further investigated especially in these identified pathways.

**Table 3:** Top 10 pathways in gene-pathway co-expression network: The maximum number of ANGPTL8 associated co-expressed genes identified in WikiPathways along with respective gene symbols are listed.

**PID**	**PathwayName**	**Gene Count**	**Genes**
WP3915	Angiopoietin Like Protein 8 Regulatory Pathway	13	MAPK8, CYP3A4, PIK3R2, PIK3R4, ANGPTL8, PRKAB2, G6PC, MAP3K11, FLOT1, PIK3C2G, SHC1, SLC16A2, RAPGEF1
WP306	Focal Adhesion	13	MAPK8, COL1A1, ACTN1, COL5A1, MET, PIK3R2, PIK3R4, ZYX, SHC1, COL3A1, PIP5K1C, ARHGAP5, RAPGEF1
WP2882	Nuclear Receptors Meta-Pathway	12	FGD4, SLCO1B1, UGT1A9, CYP3A4, ABCB11, BHLHE40, GCLM, PRDX6, BAAT, PPARGC1A, SLC19A2, IGFBP1
WP3888	VEGFA-VEGFR2 Signaling Pathway	11	MAPK8, FRS2, ATF6, PFN1, SHC1, PLCG1, PIK3R2, MYH9, GIPC1, RAPGEF1, CYP2C8
WP481	Insulin Signaling	10	PIK3C2G, MAPK8, INPP4A, SHC1, GYS2, PIK3R2, PIK3R4, RAPGEF1, MAP3K11, FLOT1
WP3362	Chromatin modifying enzymes	10	KDM6A, CARM1, SETD1A, SMARCA4, ELP2, TADA1, SMARCD1,CHD4, GATAD2A, JADE3
WP702	Metapathway biotransformation	10	UGT1A10, UGT1A9, CYP3A4, FMO4, GLYAT, BAAT, CYP4V2, HNMT, CYP2C8, NAA40
WP2857	Mesodermal Commitment Pathway	9	BMPR2, MBTD1, KDM6A, DIP2A, BHLHE40, EPB41L5, C9orf72, HPRT1, AXIN1
WP3932	Focal Adhesion-PI3K-Akt- mTOR-signaling pathway	9	COL1A1, COL5A1, COL3A1, MET,GYS2, PIK3R2, PIK3R4, GNG7,PPARGC1A
WP3925	Amino Acid metabolism	9	CTH, MAOA, FH, GCLM, MCCC1, MUT, BHMT, HNMT, AUH

Mainly Angiopoietin Like Protein 8 Regulatory Pathway, Focal Adhesion pathway, VEGFA-VEGFR2 signaling pathway and Focal Adhesion-PI3K-Akt-mTOR-signaling pathway are part of overlapping signaling pathways. Other five pathways (Amino Acid metabolism, Mesodermal Commitment pathway, Metapathway biotransformation, Chromatin modifying enzymes and Nuclear Receptors Meta-Pathway) also share several genes among them and are in line with the previously demonstrated roles of ANGPTL8 in metabolism and cell differentiation/division (Kristina and Egon, 2018; Nathan et al., 2018; Pieter et al., 2018; Reactome and Martina, 2018; Rianne et al., 2018). Overall, the results of the pathway analysis identifies important signaling pathways and associated co-expressed genes with ANGPTL8 which should be investigated further for their mutual and individual role in pathogenesis of DM and related metabolic disorders.

### 3.4. Single Nucleotide Polymorphism (SNP) Analysis

We performed a SNP identification analysis on the 72 genes present in the 10 highly associated biological processes with ANGPTL8. In Data Sheet 6 we listed both the gene names queried in the DisGeNET database associated with the top diseases, and the SNPs, located in those genes, that reported the highest DisGeNET score with the disease association. Ten genes were found associated with DM Non-Insulin Dependent (ANGPTL8, BHMT, GATAD2A, GCLM, PIK3C2G, PPARGC1A, PRKAB2, RAPGEF1, SLC19A2, and TADA1). In particular, RAPGEF1 with the intronic SNP rs11243444 (Hong et al., 2009) and PIK3C2G with the two intronic SNPs rs10841048 and rs12816270 (Daimon et al., 2008) reported association with DM Non-Insulin Dependent, PPARGC1A has the upstream SNP rs590183 associated with blood pressure (O'donnell et al., 2007) and ANGPTL8 shows the missense variant rs2278426 associated with high density lipoprotein measurement (Weissglas-Volkov et al., 2013). In addition, the SHC1 gene does not show a gene association with DM, but it has the SNP rs8191979 associated to DM (Almind et al., 1999). Moreover, GYS2 with its missense SNP rs121918420 (Orho et al., 1998), and G6PC with another missense SNP rs1801175 (Froissart et al., 2011) are associated with Glycogen storage disease type 1 (GSD1) that is a disorder characterized by severe fasting hypoglycaemia. Although, GSD1 seems completely the opposite disorder of DM, they share similar metabolic pathways leading to nephropathy and fatty liver (Rajas et al., 2013). The genes involved in the control of glucose and energy homeostasis are the same and for this reason investigating their variants effect can help to a better understanding of the role of these genes. In the Figure 3 10 genes reporting DM association and two genes associated with GSD1 are highlighted in dark blue and light blue, respectively. Moreover, when they present a SNP associated with DM or related phenotypic condition, the genes nodes are represented with triangles. The network in the Figure 3 visualizes the gene-pathway relationships, allowing to investigate deeply the roles of the seven genes already mentioned with a relevant SNP-disease association. We observed that those seven genes were grouped around five processes: Angiopoietin like protein 8 regulatory pathway (WP3915), Insulin signaling pathway (WP481), Focal adhesion-PI3K-Akt-mTOR signaling pathway (WP3932), VEGFA-VEGFR2 signaling pathway (WP3888) and Nuclear receptor meta-pathway (WP2882). The first three pathways not only contain at least one of the seven genes, but also share one or more of them. This is also due to the fact that Angiopoietin like protein 8 regulatory pathway diagram present subpaths of the other two processes, confirming the tightly biological interconnectivity within the three pathways.

## 4. Discussion

Several studies have demonstrated the role of ANGPTL8 in lipid metabolism through LPL inhibition, regulation of autophagy and adipogenesis (Ren et al., 2012; Zhang, 2012, 2016; Tseng et al., 2014a). It has also been demonstrated to regulate a crucial gene circuit required for maintenance of glucose homeostasis (Guo et al., 2016). These unique features of ANGPTL8 in regulation of different aspects of metabolism is driving the notion of its potential as a molecular target for treatment of DM. However, due to the lack of knowledge regarding its gene/protein partners, the associated biological processes and its mechanism of action, it has remained elusive to understand its role in pathogenesis of DM and subsequent assessment as molecular target. In this study, an integrated network analysis work flow especially suitable for such problems was designed to allow the extraction of relevant information with several regulatory levels. It helped us to identify the co-expressed genes with ANGPTL8, their identification as hub/nonhub genes, their presence in pathways and their co-occurrence in DM.

**Figure 4:**
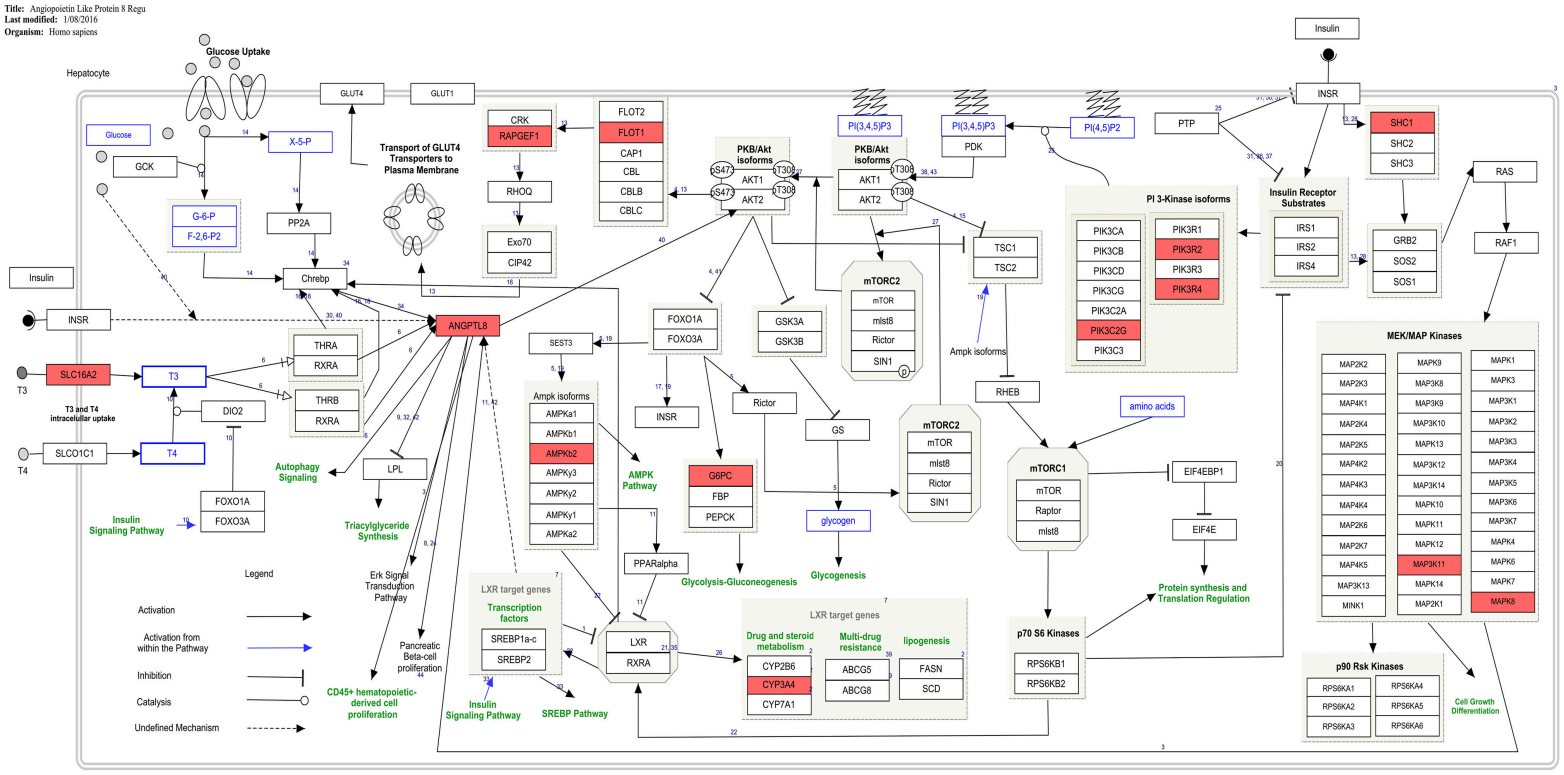
Presence of co-expressed genes of ANGPTL8 in the ANGPTL8 Regulatory Pathway (pathway id:WP3915 (www.wikipathways.org/instance/WP3915)). Thirteen genes in the ANGPTL8 regulatory pathway which are also identified to be co-expressed with it are marked with red color. These genes (MAPK8, CYP3A4, PIK3R2, PIK3R4, PRKAB2, ANGPTL8, G6PC, MAP3K11, FLOT1, PIK3C2G, SHC1, SLC16A2, RAPGEF1) belong to different signaling arms of the pathway. Among these genes, SHC1 and PIK3C2G are also identified as hub genes.

The current study provides the first instance of identification of co-expressed genes of ANGPTL8 by utilizing a liver transcriptomics data set with the outcomes which are in line with previous literature (Figure 4) and also unfolds several regulatory findings which could present an important resource for future investigations (Figure 3). The co-expressed genes of ANGPTL8 identified in this study (*n* = 460) provides narrowed down list of molecular targets which are either co-regulated with it and/or might be regulation partners at different levels of interaction. Current analysis revealed the co-expression of thirteen genes with ANGPTL8 in the literature curated pathway of ANGPTL8 (WP3915) which was designed in our previous work (Siddiqa et al., 2017). These findings provides support to the current analysis and also emphasizes the association of the thirteen revealed molecular players with ANGPTL8 in its pathway due to shared co-expression pattern (Figure 4).

Previous studies demonstrated the role of ANGPTL8 in several biological processes such as carbohydrate and lipid metabolism, adipogenesis, autophagy and CD45+ hematopoietic cell proliferation with none and/or partially identified partner proteins. The GO analysis performed in this study revealed several biological processes (in line with these previous literature findings) and the associated genes from co-expression network of ANGPTL8. Thus, the revealed genes in each biological process have implications for future investigation as being co-regulated with ANGPTL8 or mutual engagement in these processes. The findings of the pathway analysis in the current study provides another level of information on the role of ANGPTL8 in the identified biological processes. It allows us to view the interactions between ANGPTL8 and the co-expressed genes based on the previously identified pathway diagrams present in WikiPathways. The gene-pathway network represented in Figure 3 helped to identify visually the relationships between significant pathways and co-expressed genes with SNPs associated to DM and similar phenotypic traits. It is remarkable how the seven genes identified with a relevant SNPs association, happen to be clustered around processes linked with the Angiopoietin like protein 8 regulatory pathway. Although in some studies the variants-disease associations were detected in different populations than the Caucasian, such as Korean (Hong et al., 2009) and Aborigen (Daimon et al., 2008), the literature regarding the gene-disease associations of those genes included Caucasian individuals as well. The effect of the SNPs is not always well characterized except for the missense variant of the ANGPTL8 (Weissglas-Volkov et al., 2013). For this reason exploring the possible SNP effects in the pathways identified by those genes is not feasible with the literature information retrieved. However, from this genetic investigation it is possible to observe that there are significant genetic signals associated to DM and similar traits, influencing genes involved and co-expressed in ANGPTL8 pathways. Thus, further experimental studies on those genes need to take the genetic background into account or under control in case of mice studies. Moreover, upcoming or existing GWAS studies for DM, could be checked for signals related to the co-expressed ANGTPL8 genes, to properly assess their relevancy in the pathophysiology of the disease.

The key findings of this study provide focused information on molecular players co-expressed with ANGPTL8 and associated pathways with implications for follow up experimental studies which could aid in identifying the exact mechanism of action and signaling events leading to pathogenesis of DM and metabolic disorders. Moreover, the integrated systems biology workflow deployed in this study provides a way to assess the gene-centric insights and to elucidate different levels of regulation from a transcriptomics data, in contrast to the typical -omics workflows which less directly target the systems level knowledge.

## 5. Conclusion

In this study, an integrated systems biology workflow is deployed to analyze a hepatic transcriptomics data set. The co-expression network analysis coupled with pathways analysis of this data aided in identification of the genes associated with ANGPTL8 at different levels of regulation. The findings of GO analysis provided the complete annotation of the ANGPTL8 co-expression genes module. Moreover, the genes already associated with DM in ANGPTL8 genes co-expression network were identified which increased our knowledge regarding the possible mutual engagement of these genes in the pathogenic mechanism. All of the findings of this study have implications for follow up experimental studies which could aid in identifying the exact mechanism of action and signaling events leading to pathogenesis of DM and metabolic disorders. Moreover, the integrated analysis workflow based on different methods and tools employed in the current study allows to assess a previously less characterized or uncharacterized gene/protein in a systematic way which may aid future studies.

## Author Contributions

SC, CE, and AS conceived the experiments. AS and EC conducted the experiments. All the authors took part in discussions, analysis and layout of results and reviewed the manuscript. AS wrote the paper.

### Conflict of Interest Statement

The authors declare that the research was conducted in the absence of any commercial or financial relationships that could be construed as a potential conflict of interest.

## Footnotes

^1^https://www.wikipathways.org/index.php/Pathway:WP3915

^2^https://www.ncbi.nlm.nih.gov/geo/query/acc.cgi?acc=GSE64998

## Supplemental Data

The transcriptomic data set used for analysis was already available in the Gene Expression Omnibus database under accession no GSE6498. All the subsequent data generated and analyzed during this study are included in this published article (and its Supplementary Information Files).

Figure S1 The Gene Ontology analysis visualized as a network.

Figure S2 The co-expression network of ANGPTL8 based on the red genes module.

Figure S3 Genes to pathway network visualization of the co-expression network of ANGPTL8.

Presentation 1 The complete quality checks report generated from ArrayAnalysis.org for the microarray data before and after normalization/preprocessing is provided.

Data Sheet 1 The complete gene expression data file after performing normalization without filtering for the low expression probes.

Data Sheet 2 The normalized and preprocessed gene expressions used for WGCNA.

Data Sheet 3 The complete R Code used to perform WGCNA.

Data Sheet 4 List of genes to SNPs analysis performed on the selection of genes from ANGPTL8 genes co-expression module.

Data Sheet 5 Lists of the genes in corresponding modules identified through WGCNA.

Data Sheet 6 The Gene Ontology analysis of the ANGPTL8 associated genes co-expression module.

Data Sheet 7 List of pathways enlisted with the WikiPathway IDs and corresponding identified genes from ANGPTL8 co-expression genes module in them.
