# Supplementary figures and images for: Biological Pathways Leading From ANGPTL8 to Diabetes Mellitus–A Co-expression Network Based Analysis
Source: Front Physiol. 2018 Dec 21;9:1841. doi: 10.3389/fphys.2018.01841 (PMC6309236; doi:10.3389/fphys.2018.01841)

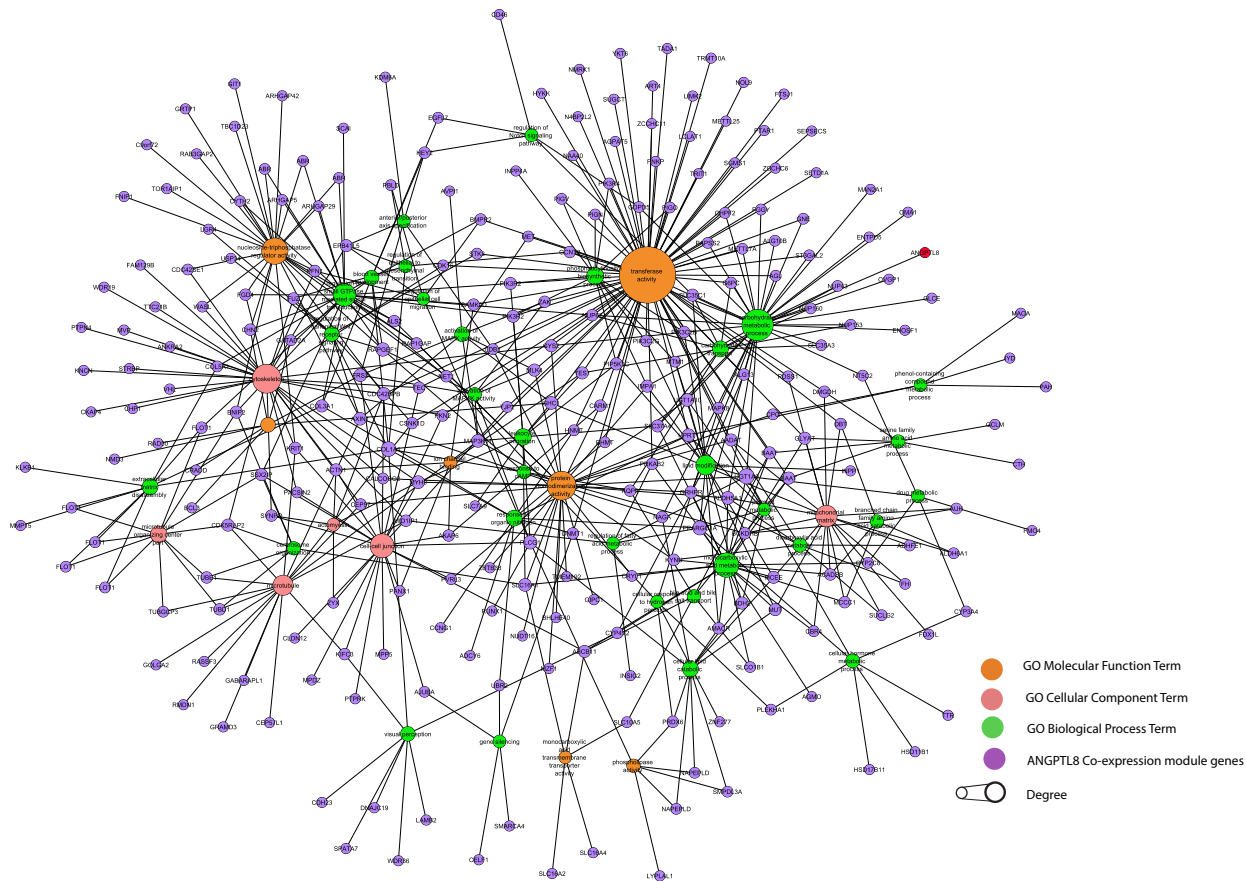

Supplement: Figure S1 — The Gene Ontology analysis visualized as a network. [file Image_1.PDF]

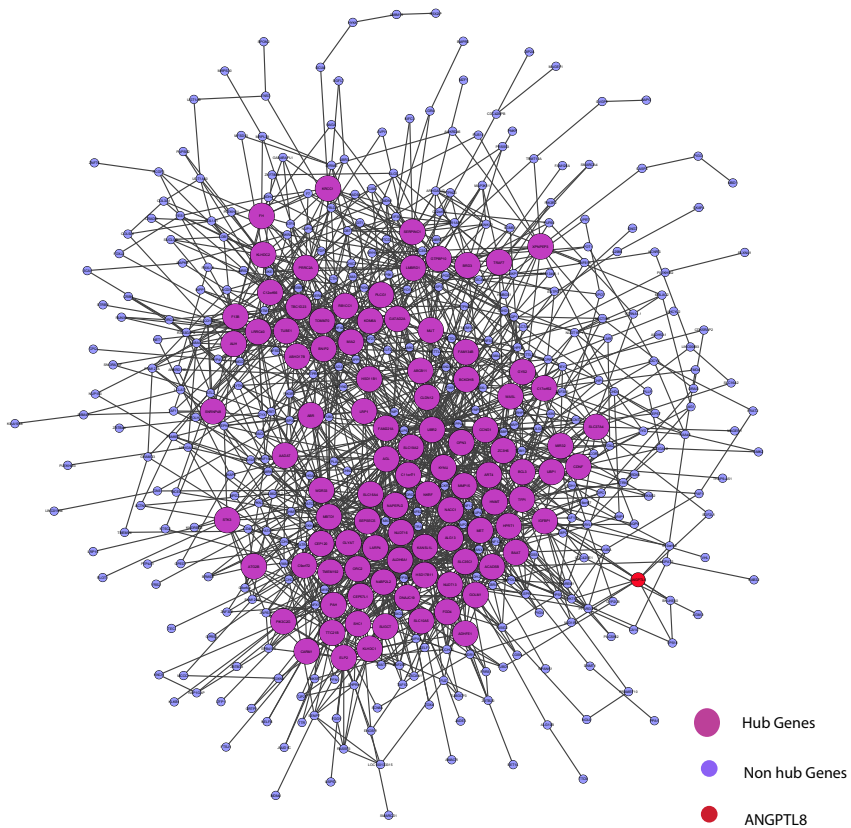

Supplement: Figure S2 — The co-expression network of ANGPTL8 based on the red genes module. [file Image_2.pdf]

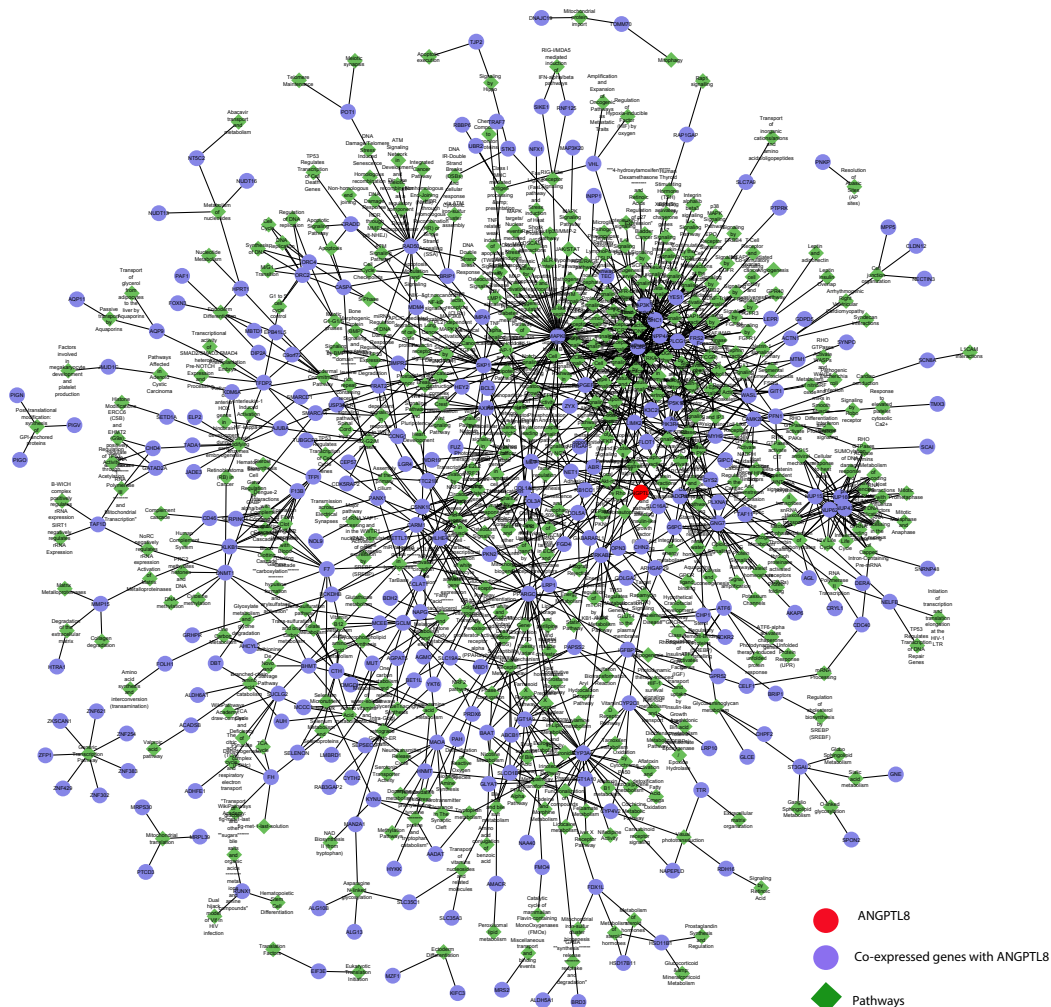

Supplement: Figure S3 — Genes to pathway network visualization of the co-expression network of ANGPTL8. [file Image_3.pdf]
