# Supplementary material for: Biological Pathways Leading From ANGPTL8 to Diabetes Mellitus–A Co-expression Network Based Analysis
Source: Front Physiol. 2018 Dec 21;9:1841. doi: 10.3389/fphys.2018.01841 (PMC6309236; doi:10.3389/fphys.2018.01841)
Supplement: Presentation 1 — The complete quality checks report generated from ArrayAnalysis.org for the microarray data before and after normalization/preprocessing is provided. [file Presentation_1.PDF]

Quality Control & Pre-processing Evaluation

of

GSE64998_0

REPORT

Array names and grouping

ArrayDataFile	SourceName	FactorValue
GSM1585583.CEL	obese-ND-rep7	obese-ND
GSM1585584.CEL	obese-ND-rep8	obese-ND
GSM1585585.CEL	obese-T2D-rep1	obese-T2D
GSM1585586.CEL	obese-T2D-rep2	obese-T2D
GSM1585587.CEL	obese-T2D-rep3	obese-T2D
GSM1585588.CEL	obese-T2D-rep4	obese-T2D
GSM1585589.CEL	obese-T2D-rep5	obese-T2D
GSM1585590.CEL	obese-T2D-rep6	obese-T2D
GSM1585591.CEL	obese-T2D-rep7	obese-T2D
GSM1585592.CEL	nonobese-rep1	nonobese
GSM1585593.CEL	nonobese-rep2	nonobese
GSM1585594.CEL	nonobese-rep3	nonobese
GSM1585595.CEL	nonobese-rep4	nonobese
GSM1585596.CEL	nonobese-rep5	nonobese
GSM1585597.CEL	nonobese-rep6	nonobese
GSM1585577.CEL	obese-ND-rep1	obese-ND
GSM1585578.CEL	obese-ND-rep2	obese-ND
GSM1585579.CEL	obese-ND-rep3	obese-ND
GSM1585580.CEL	obese-ND-rep4	obese-ND
GSM1585581.CEL	obese-ND-rep5	obese-ND
GSM1585582.CEL	obese-ND-rep6	obese-ND

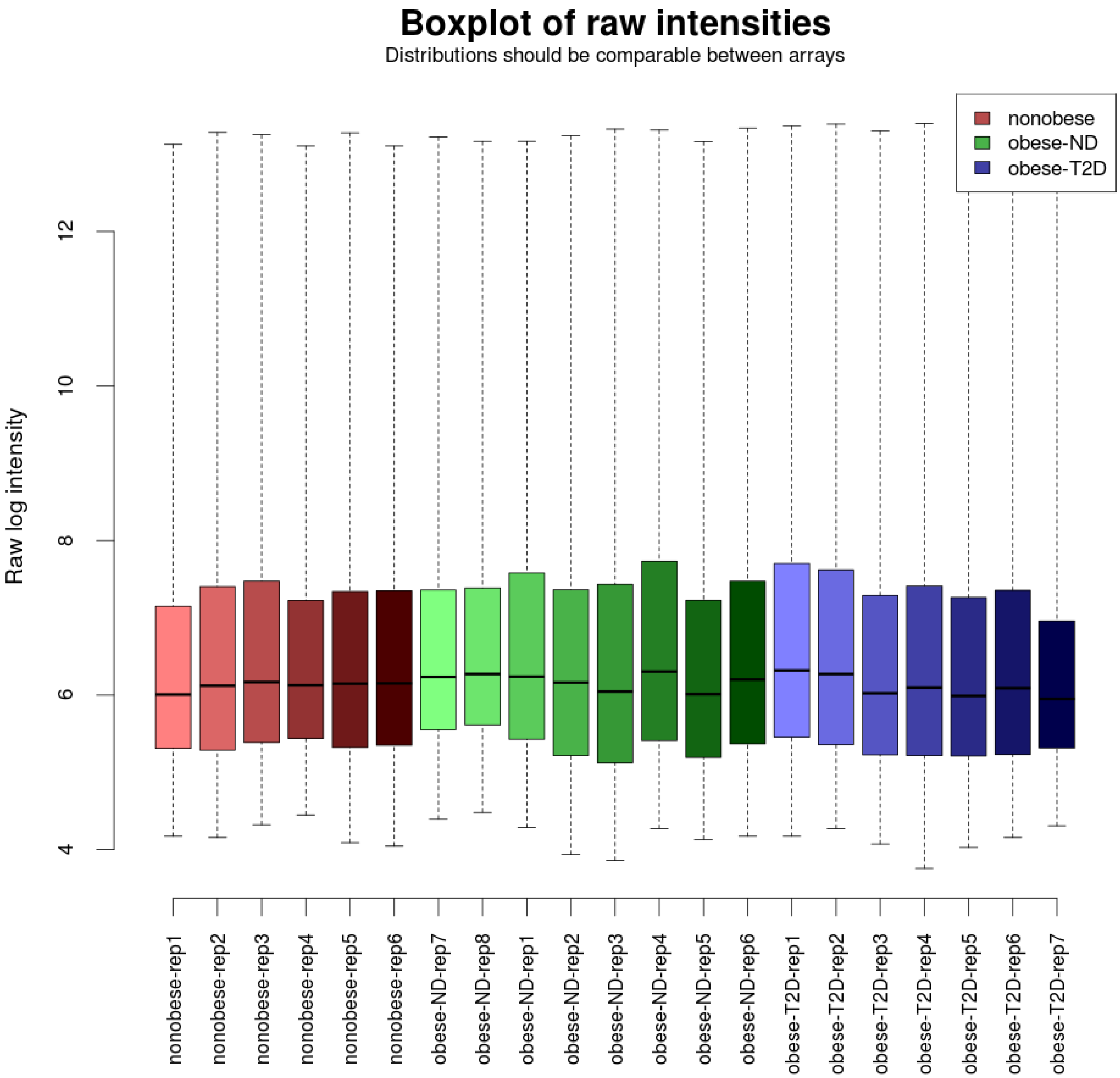

Boxplot of raw intensities
Distributions should be comparable between arrays
nonobese
obese-ND
obese-T2D
Raw log intensity
4
6
8
10
12
nonobese-rep1
nonobese-rep2
nonobese-rep3
nonobese-rep4
nonobese-rep5
nonobese-rep6
obese-ND-rep7
obese-ND-rep8
obese-ND-rep1
obese-ND-rep2
obese-ND-rep3
obese-ND-rep4
obese-ND-rep5
obese-ND-rep6
obese-T2D-rep1
obese-T2D-rep2
obese-T2D-rep3
obese-T2D-rep4
obese-T2D-rep5
obese-T2D-rep6
obese-T2D-rep7

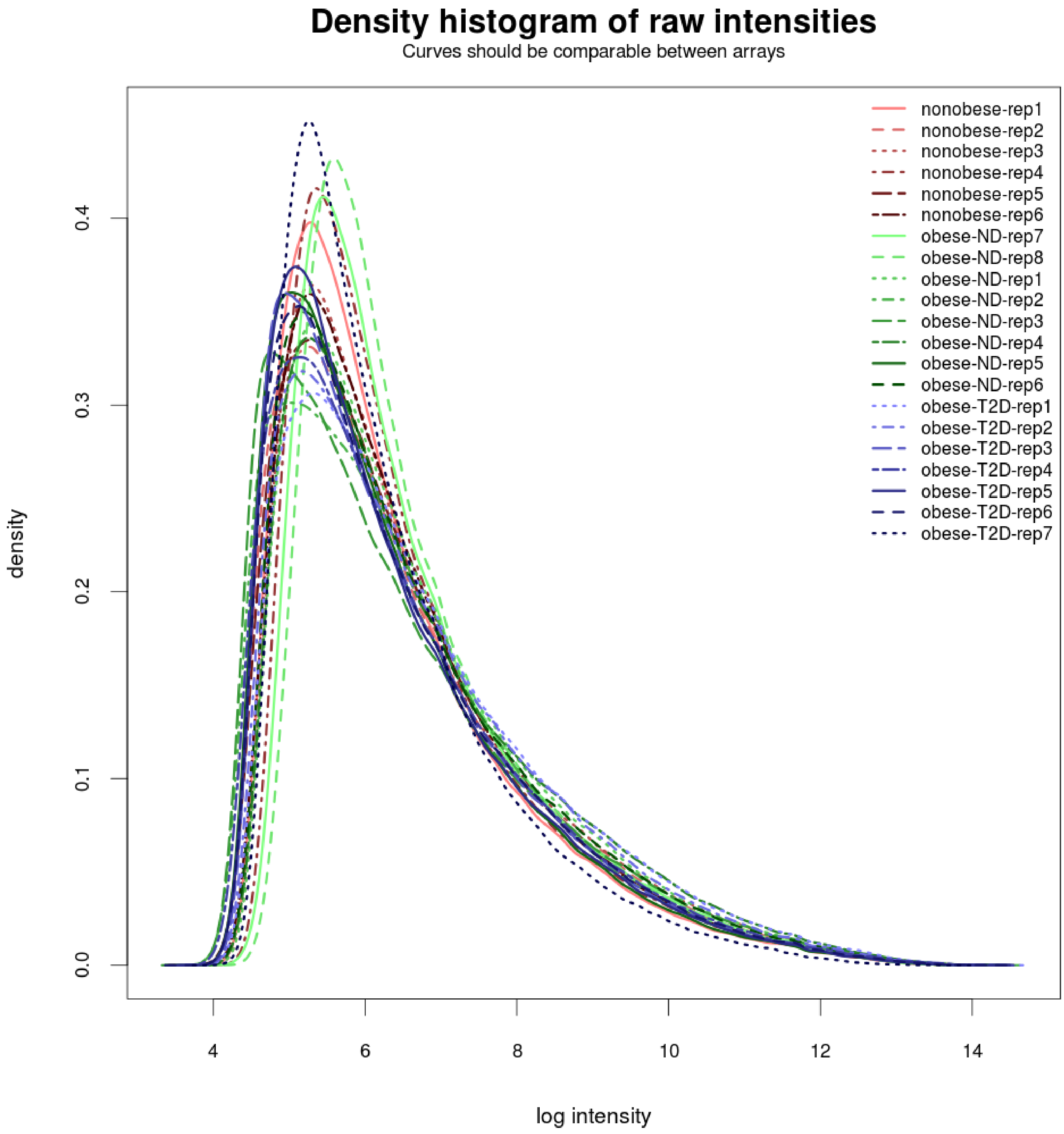
Density histogram of raw intensities
Curves should be comparable between arrays
density
log intensity
0.0
0.1
0.2
0.3
0.4
4
6
8
10
12
14
nonobese-rep1
nonobese-rep2
nonobese-rep3
nonobese-rep4
nonobese-rep5
nonobese-rep6
obese-ND-rep7
obese-ND-rep8
obese-ND-rep1
obese-ND-rep2
obese-ND-rep3
obese-ND-rep4
obese-ND-rep5
obese-ND-rep6
obese-T2D-rep1
obese-T2D-rep2
obese-T2D-rep3
obese-T2D-rep4
obese-T2D-rep5
obese-T2D-rep6
obese-T2D-rep7

MA plots of raw data 1 / 2

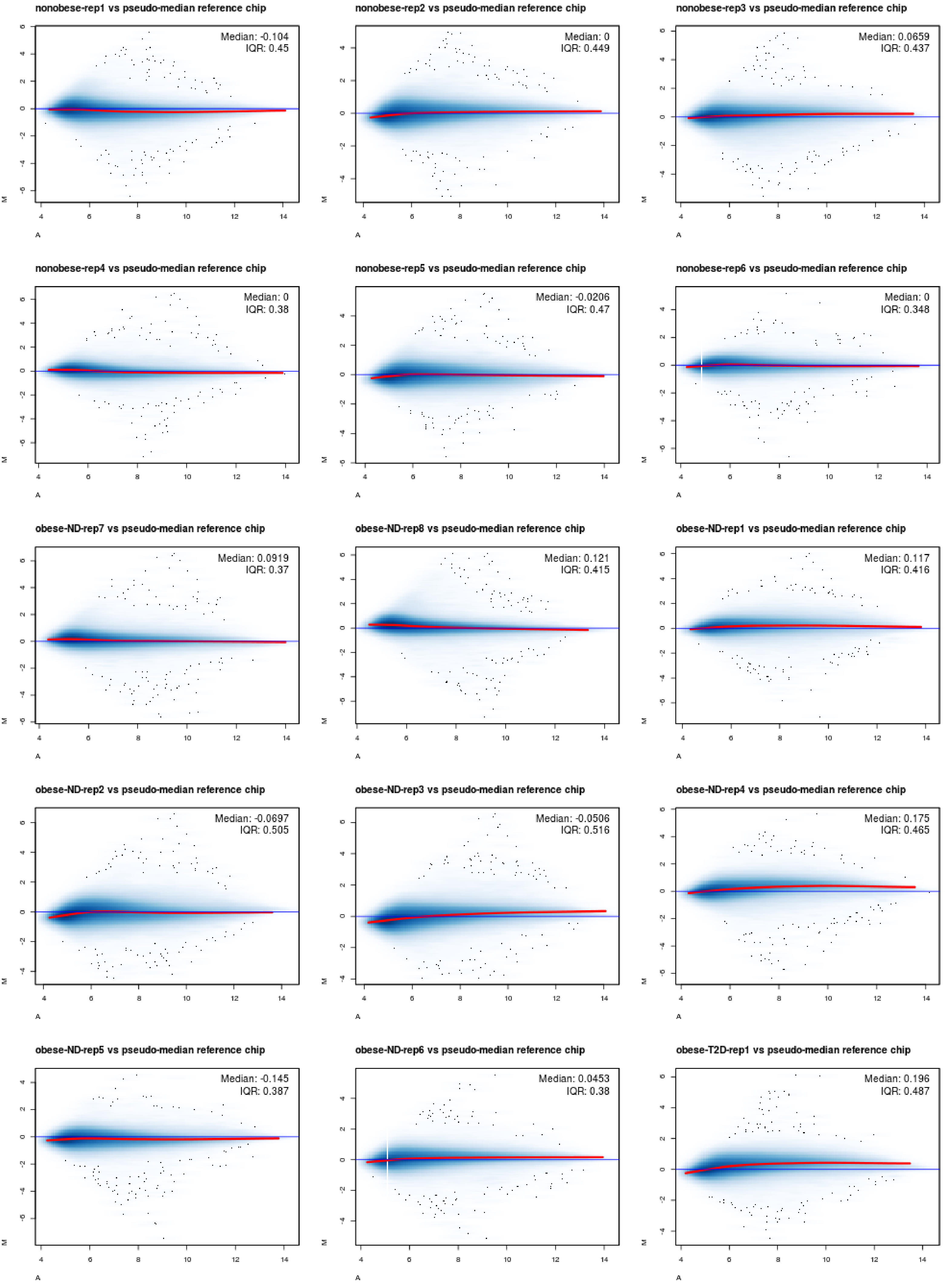

MA plots of raw data 2 / 2

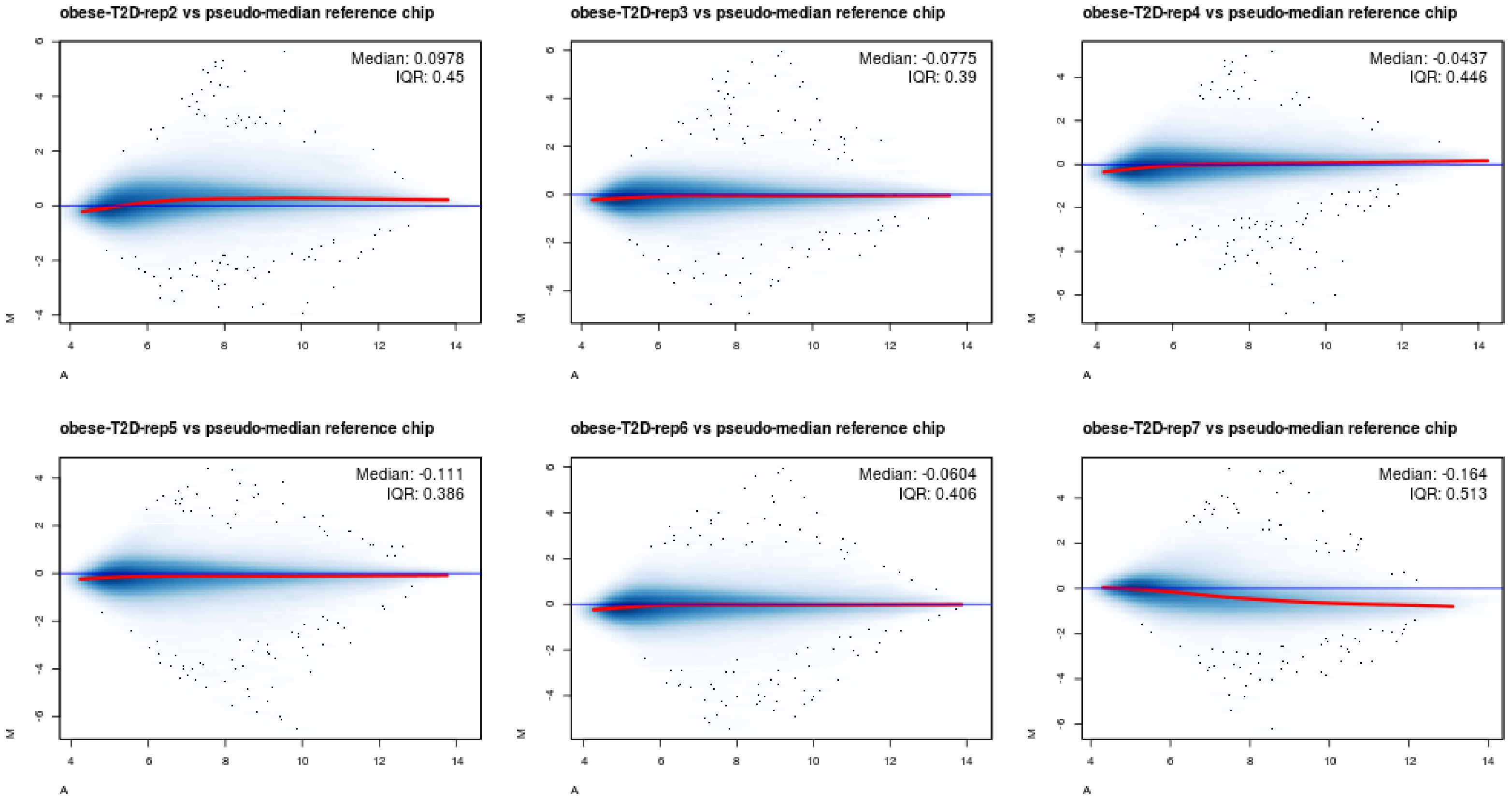

2D virtual PLM image for model characteristic: resids 1 / 2

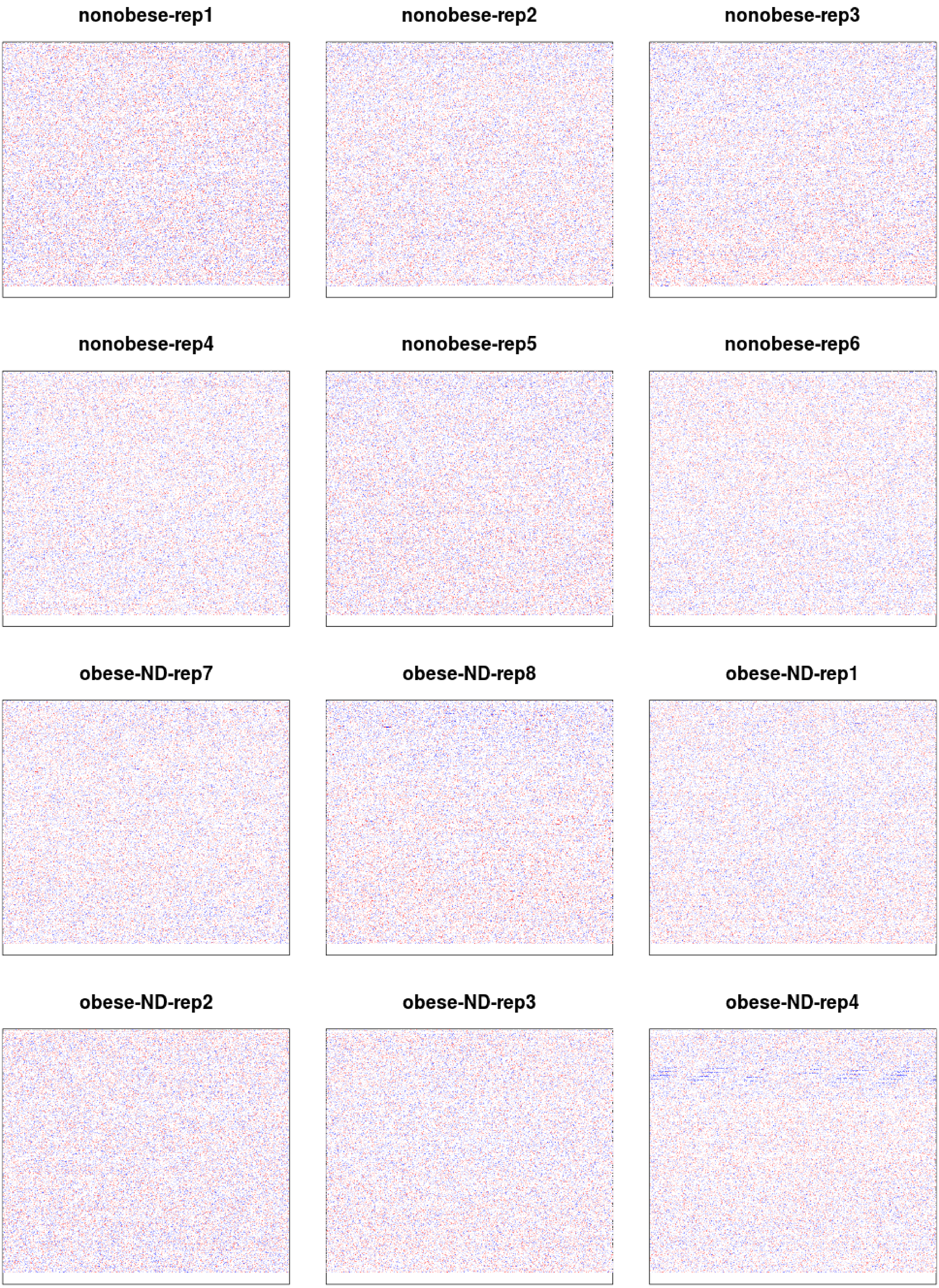

2D virtual PLM image for model characteristic: resids 2 / 2

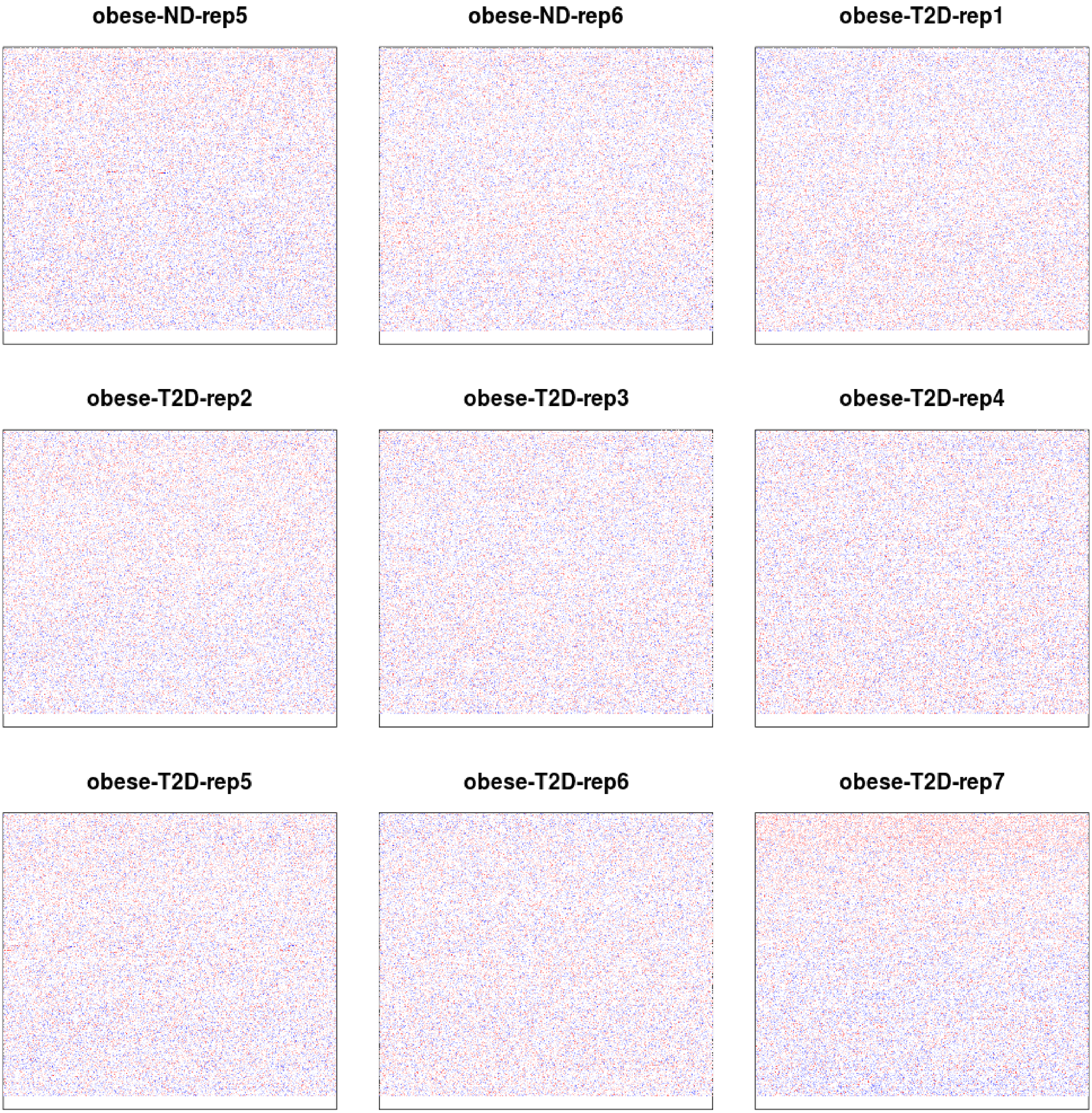

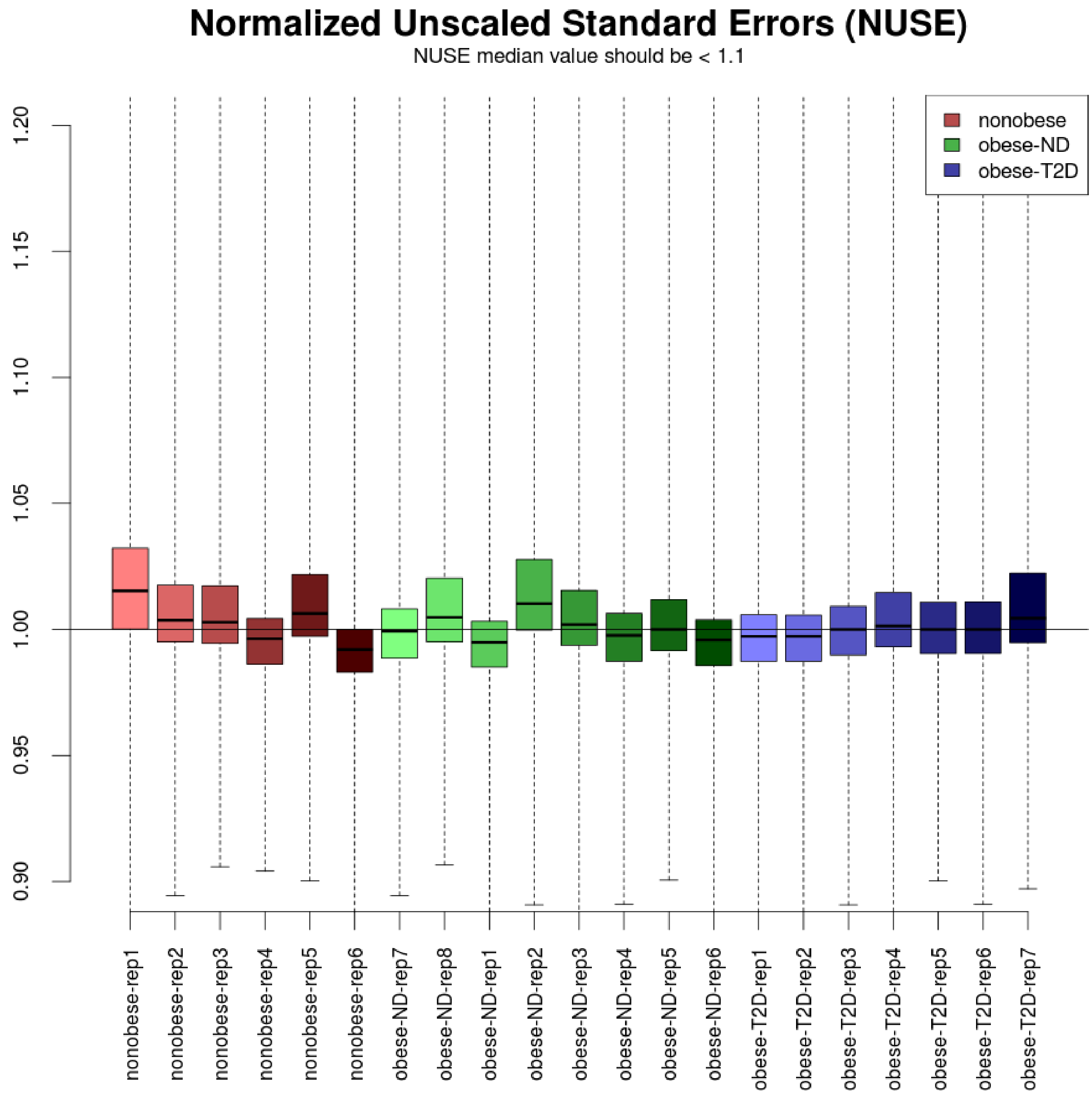
Normalized Unscaled Standard Errors (NUSE)
NUSE median value should be < 1.1
nonobese
obese-ND
obese-T2D
1.20
1.15
1.10
1.05
1.00
0.95
0.90
nonobese-rep1
nonobese-rep2
nonobese-rep3
nonobese-rep4
nonobese-rep5
nonobese-rep6
obese-ND-rep7
obese-ND-rep8
obese-ND-rep1
obese-ND-rep2
obese-ND-rep3
obese-ND-rep4
obese-ND-rep5
obese-ND-rep6
obese-T2D-rep1
obese-T2D-rep2
obese-T2D-rep3
obese-T2D-rep4
obese-T2D-rep5
obese-T2D-rep6
obese-T2D-rep7

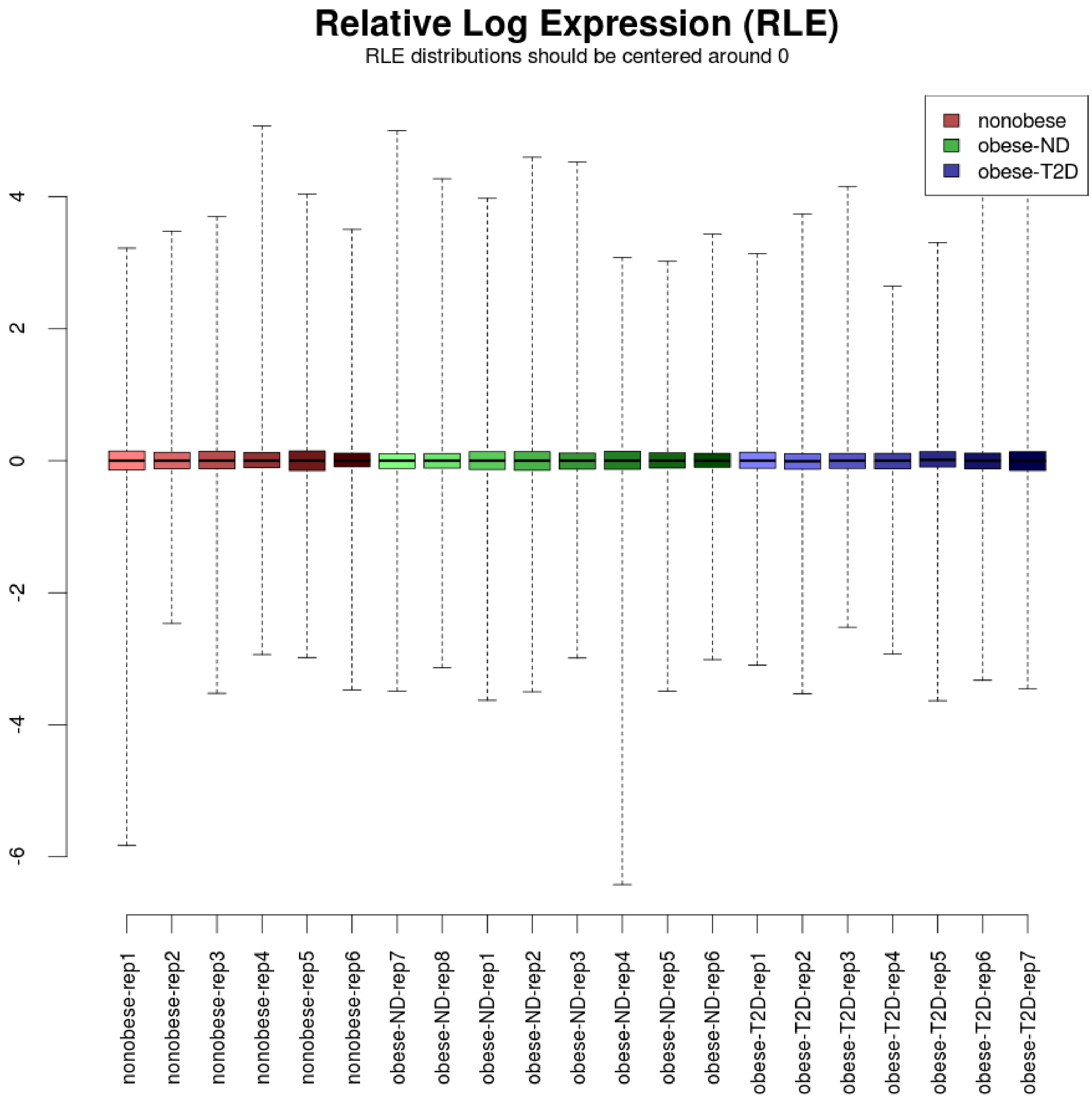

Relative Log Expression (RLE)
RLE distributions should be centered around 0
nonobese
obese-ND
obese-T2D
4
2
0
-2
-4
-6
nonobese-rep1
nonobese-rep2
nonobese-rep3
nonobese-rep4
nonobese-rep5
nonobese-rep6
obese-ND-rep7
obese-ND-rep8
obese-ND-rep1
obese-ND-rep2
obese-ND-rep3
obese-ND-rep4
obese-ND-rep5
obese-ND-rep6
obese-T2D-rep1
obese-T2D-rep2
obese-T2D-rep3
obese-T2D-rep4
obese-T2D-rep5
obese-T2D-rep6
obese-T2D-rep7

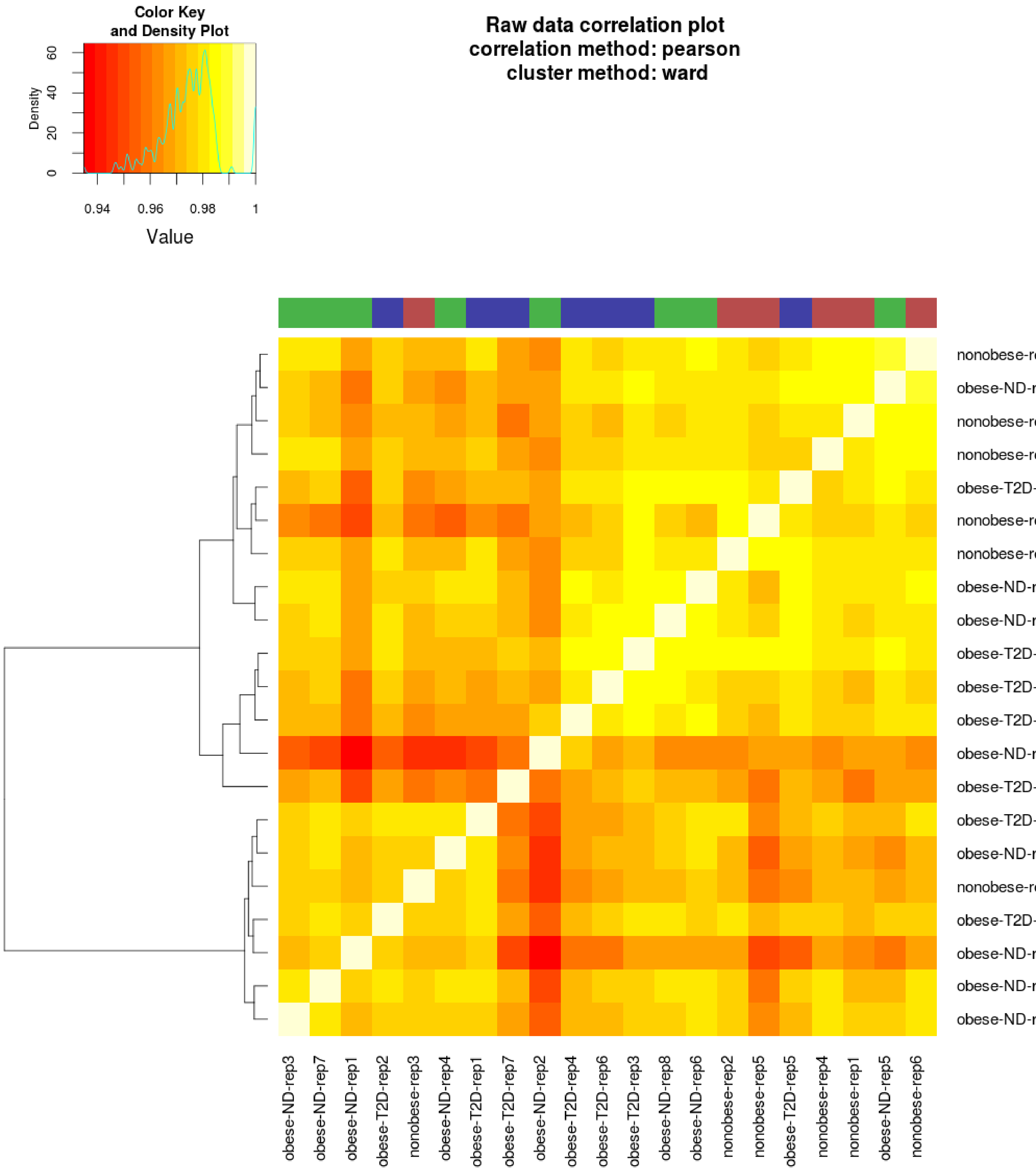

Color Key
and Density Plot
Density
Value
0.94
0.96
0.98
1
0
20
40
60
Raw data correlation plot
correlation method: pearson
cluster method: ward
obese-ND-rep3
obese-ND-rep7
obese-ND-rep1
obese-T2D-rep2
nonobese-rep3
obese-ND-rep4
obese-T2D-rep1
obese-T2D-rep7
obese-ND-rep2
obese-T2D-rep4
obese-T2D-rep6
obese-T2D-rep3
obese-ND-rep8
obese-ND-rep6
nonobese-rep2
nonobese-rep5
obese-T2D-rep5
nonobese-rep4
nonobese-rep1
obese-ND-rep5
nonobese-rep6

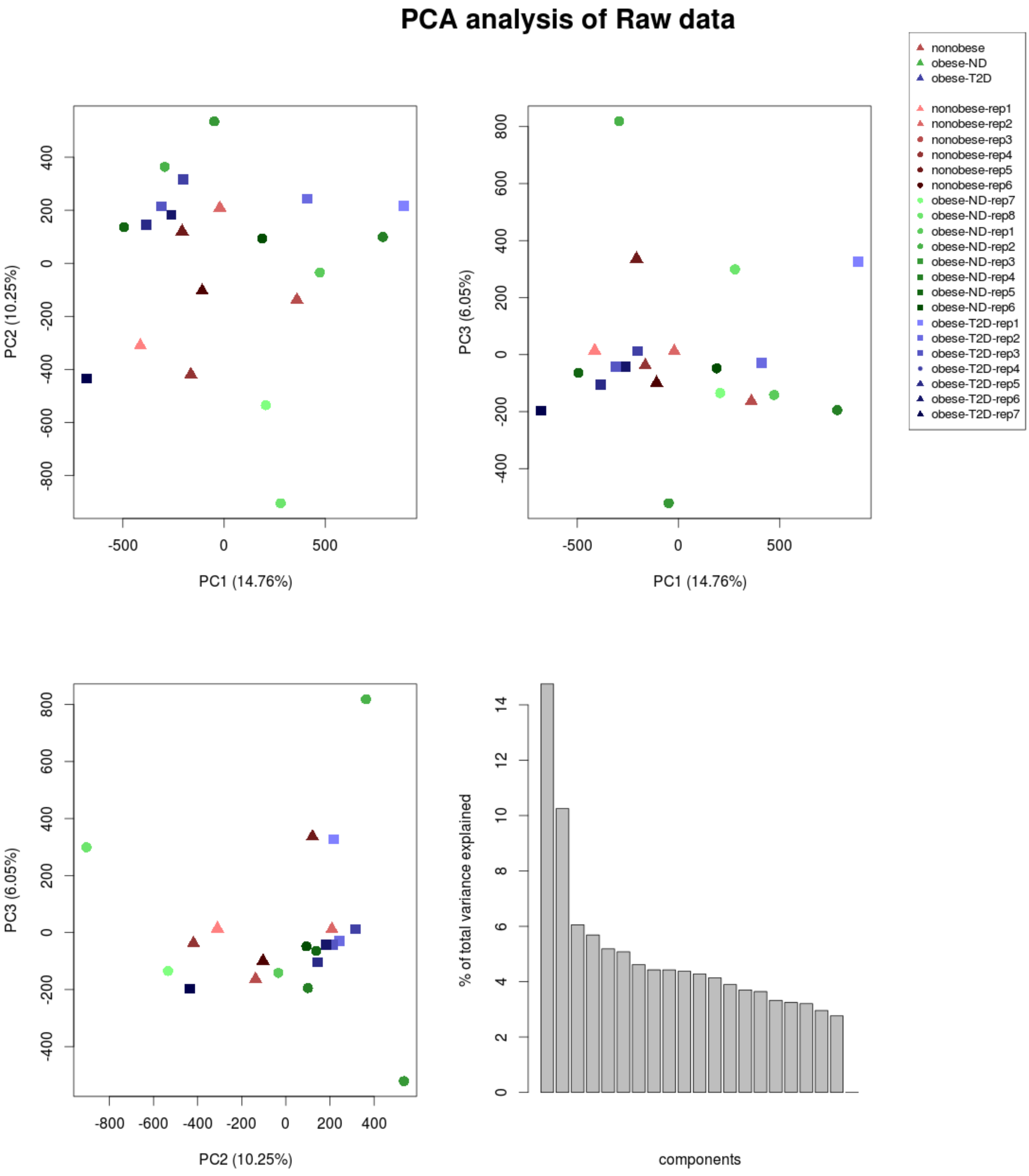
PCA analysis of Raw data
PC1 (14.76%)
PC2 (10.25%)
PC3 (6.05%)
% of total variance explained
components
nonobese
obese-ND
obese-T2D
nonobese-rep1
nonobese-rep2
nonobese-rep3
nonobese-rep4
nonobese-rep5
nonobese-rep6
obese-ND-rep7
obese-ND-rep8
obese-ND-rep1
obese-ND-rep2
obese-ND-rep3
obese-ND-rep4
obese-ND-rep5
obese-ND-rep6
obese-T2D-rep1
obese-T2D-rep2
obese-T2D-rep3
obese-T2D-rep4
obese-T2D-rep5
obese-T2D-rep6
obese-T2D-rep7

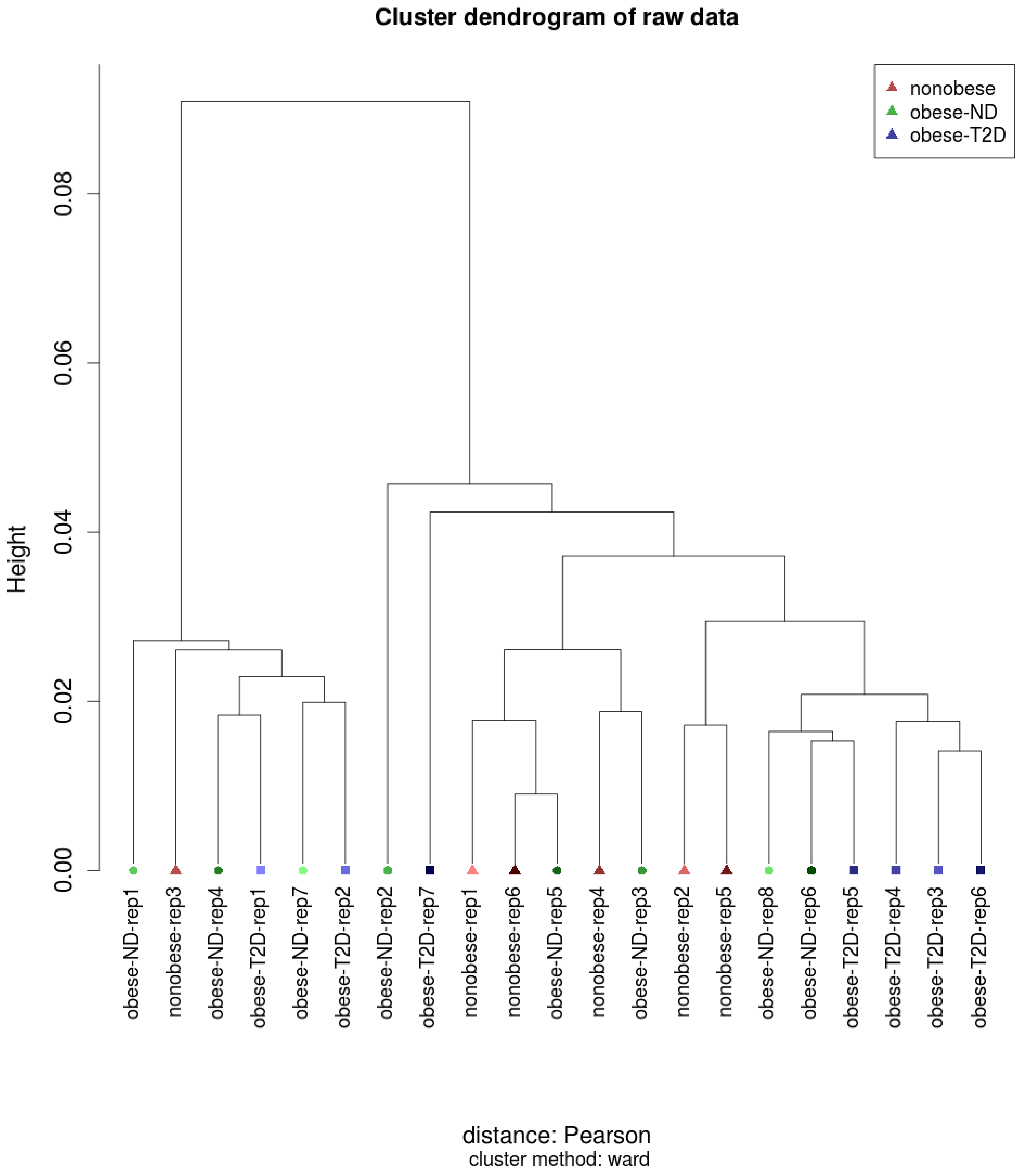
Cluster dendrogram of raw data
nonobese
obese-ND
obese-T2D
Height
0.00
0.02
0.04
0.06
0.08
obese-ND-rep1
nonobese-rep3
obese-ND-rep4
obese-T2D-rep1
obese-ND-rep7
obese-T2D-rep2
obese-ND-rep2
obese-T2D-rep7
nonobese-rep1
nonobese-rep6
obese-ND-rep5
nonobese-rep4
obese-ND-rep3
nonobese-rep2
nonobese-rep5
obese-ND-rep8
obese-ND-rep6
obese-T2D-rep5
obese-T2D-rep4
obese-T2D-rep3
obese-T2D-rep6
distance: Pearson
cluster method: ward

Pre-processing of Raw Data

Method: RMA
Annotation: hugene11st_Hs_ENTREZG

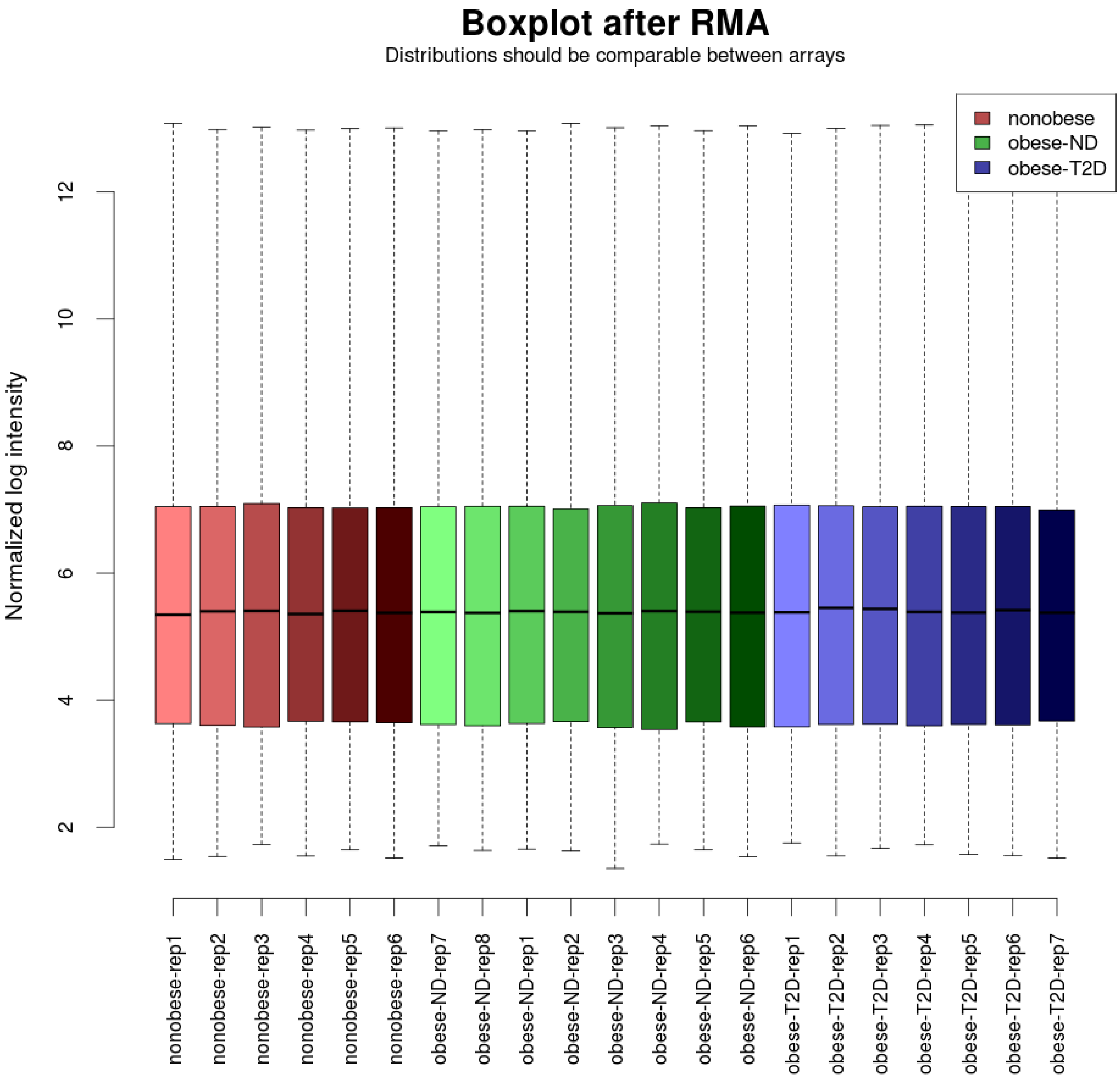
Boxplot after RMA
Distributions should be comparable between arrays
nonobese
obese-ND
obese-T2D
Normalized log intensity
2
4
6
8
10
12
nonobese-rep1
nonobese-rep2
nonobese-rep3
nonobese-rep4
nonobese-rep5
nonobese-rep6
obese-ND-rep7
obese-ND-rep8
obese-ND-rep1
obese-ND-rep2
obese-ND-rep3
obese-ND-rep4
obese-ND-rep5
obese-ND-rep6
obese-T2D-rep1
obese-T2D-rep2
obese-T2D-rep3
obese-T2D-rep4
obese-T2D-rep5
obese-T2D-rep6
obese-T2D-rep7

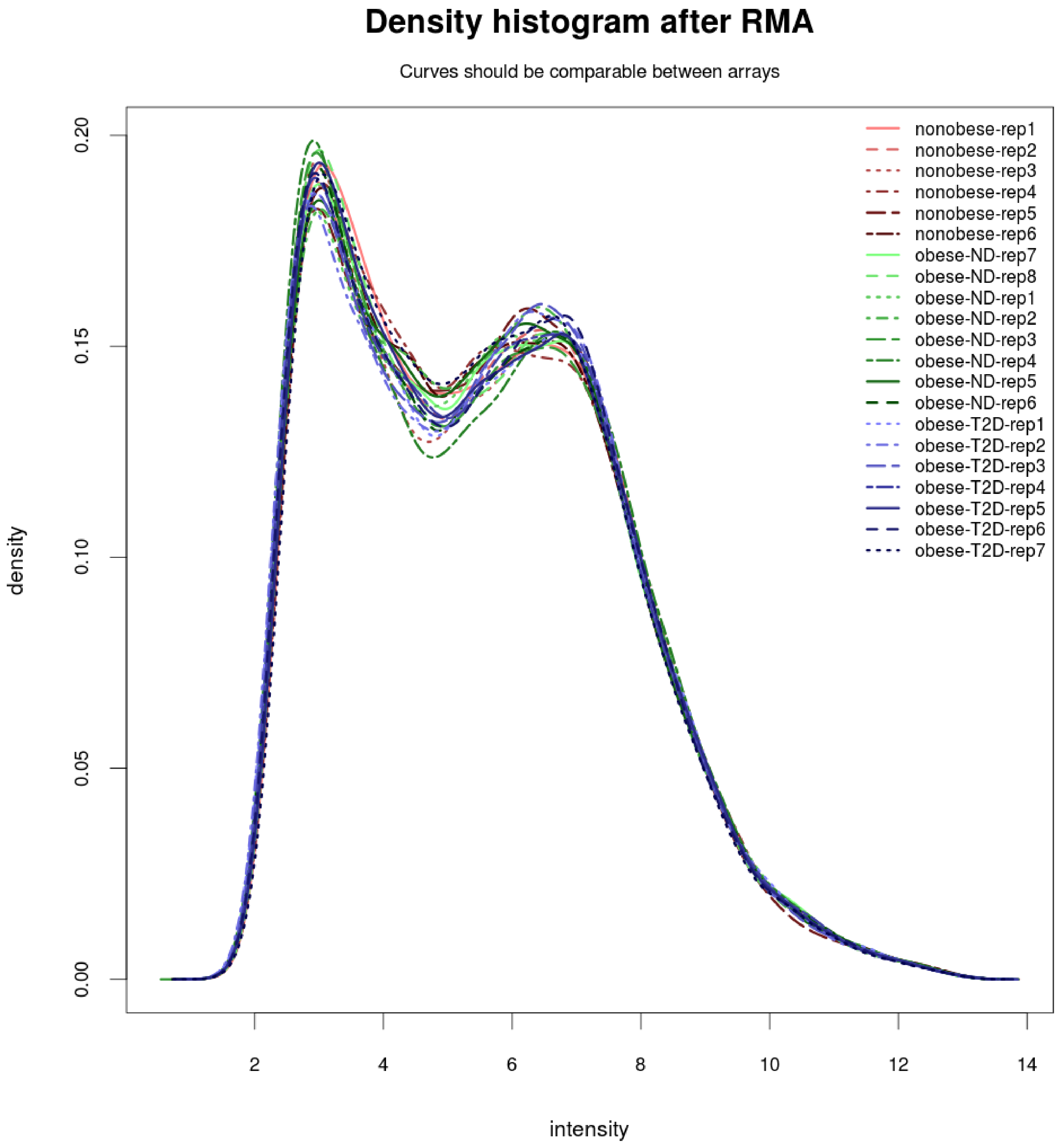
Density histogram after RMA
Curves should be comparable between arrays
density
intensity
0.00
0.05
0.10
0.15
0.20
2
4
6
8
10
12
14
nonobese-rep1
nonobese-rep2
nonobese-rep3
nonobese-rep4
nonobese-rep5
nonobese-rep6
obese-ND-rep7
obese-ND-rep8
obese-ND-rep1
obese-ND-rep2
obese-ND-rep3
obese-ND-rep4
obese-ND-rep5
obese-ND-rep6
obese-T2D-rep1
obese-T2D-rep2
obese-T2D-rep3
obese-T2D-rep4
obese-T2D-rep5
obese-T2D-rep6
obese-T2D-rep7

MA plots afterRMAnormalization 1 / 2

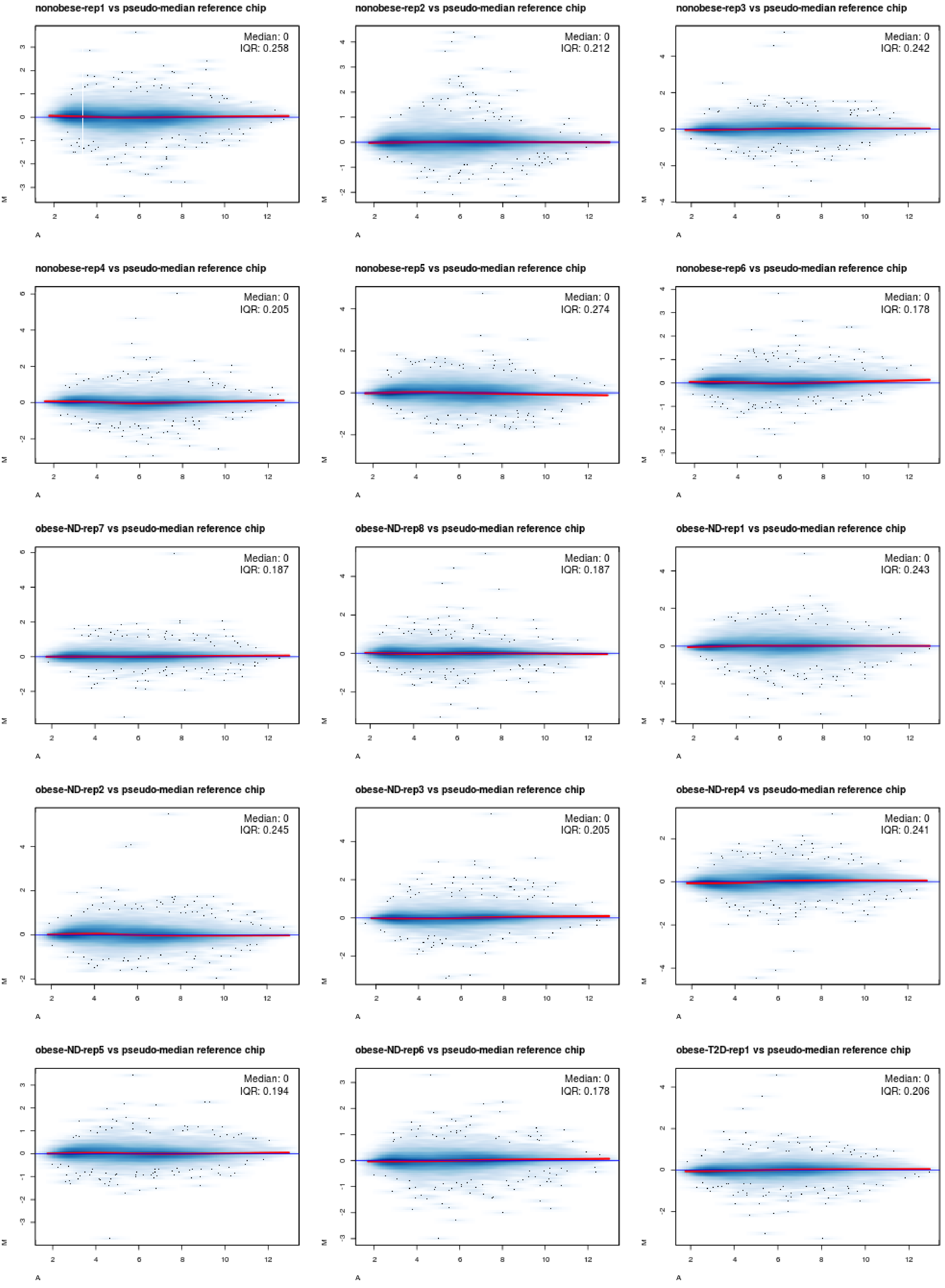

MA plots afterRMAnormalization 2 / 2

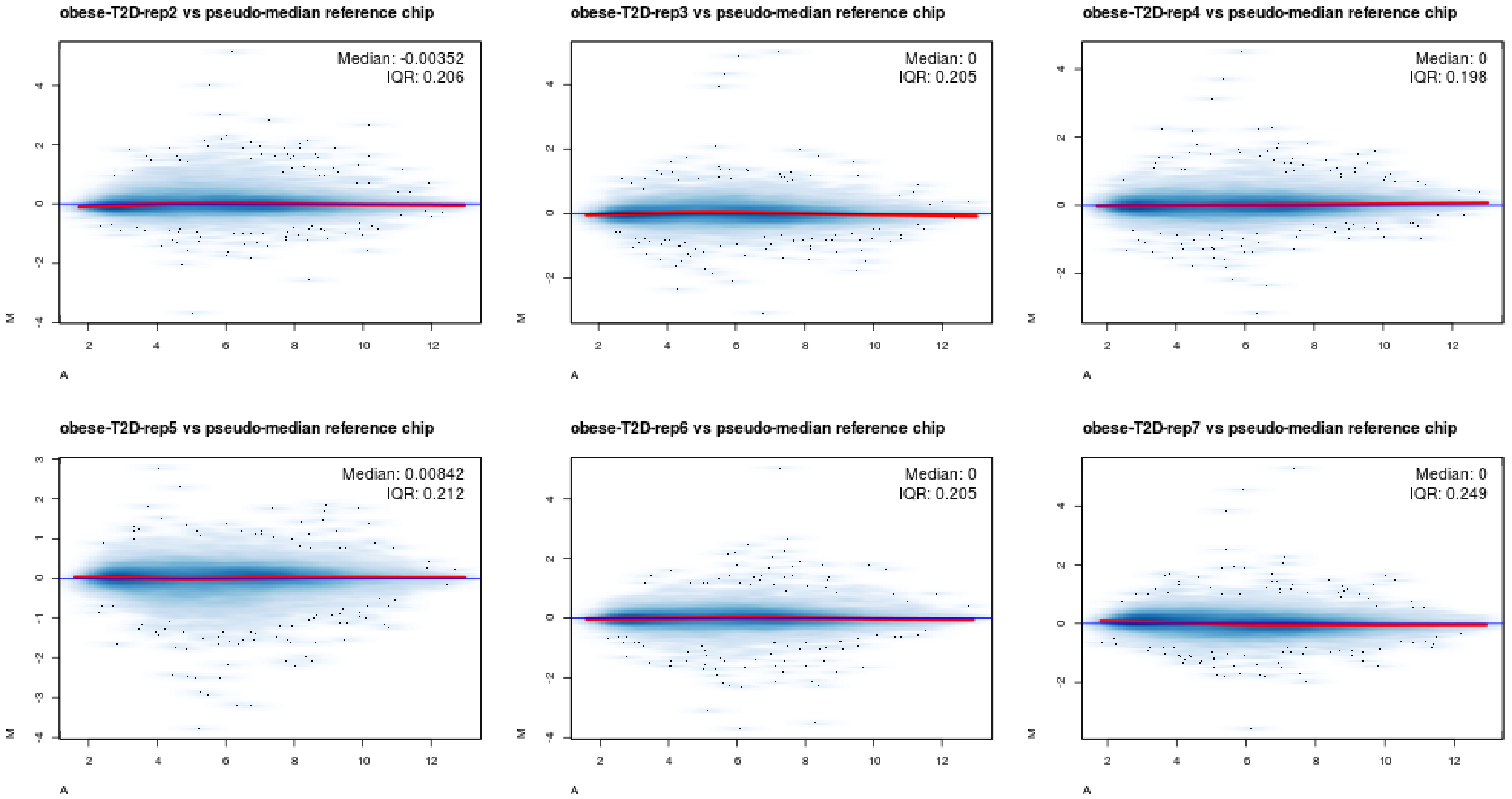

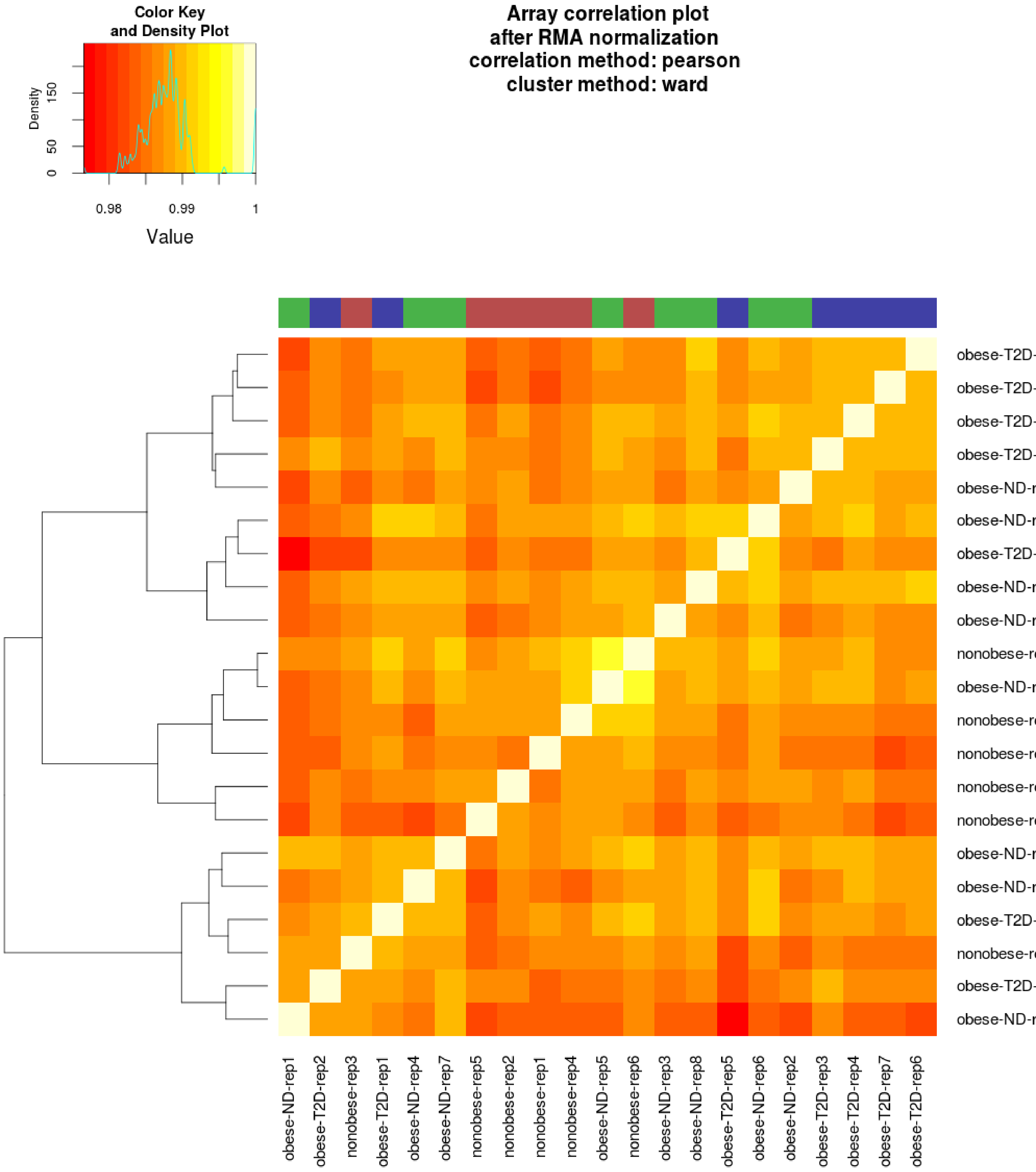
Color Key
and Density Plot
Density
0
50
150
0.98
0.99
1
Value
Array correlation plot
after RMA normalization
correlation method: pearson
cluster method: ward
obese-ND-rep1
obese-T2D-rep2
nonobese-rep3
obese-T2D-rep1
obese-ND-rep4
obese-ND-rep7
nonobese-rep5
nonobese-rep2
nonobese-rep1
nonobese-rep4
obese-ND-rep5
nonobese-rep6
obese-ND-rep3
obese-ND-rep8
obese-T2D-rep5
obese-ND-rep6
obese-ND-rep2
obese-T2D-rep3
obese-T2D-rep4
obese-T2D-rep7
obese-T2D-rep6

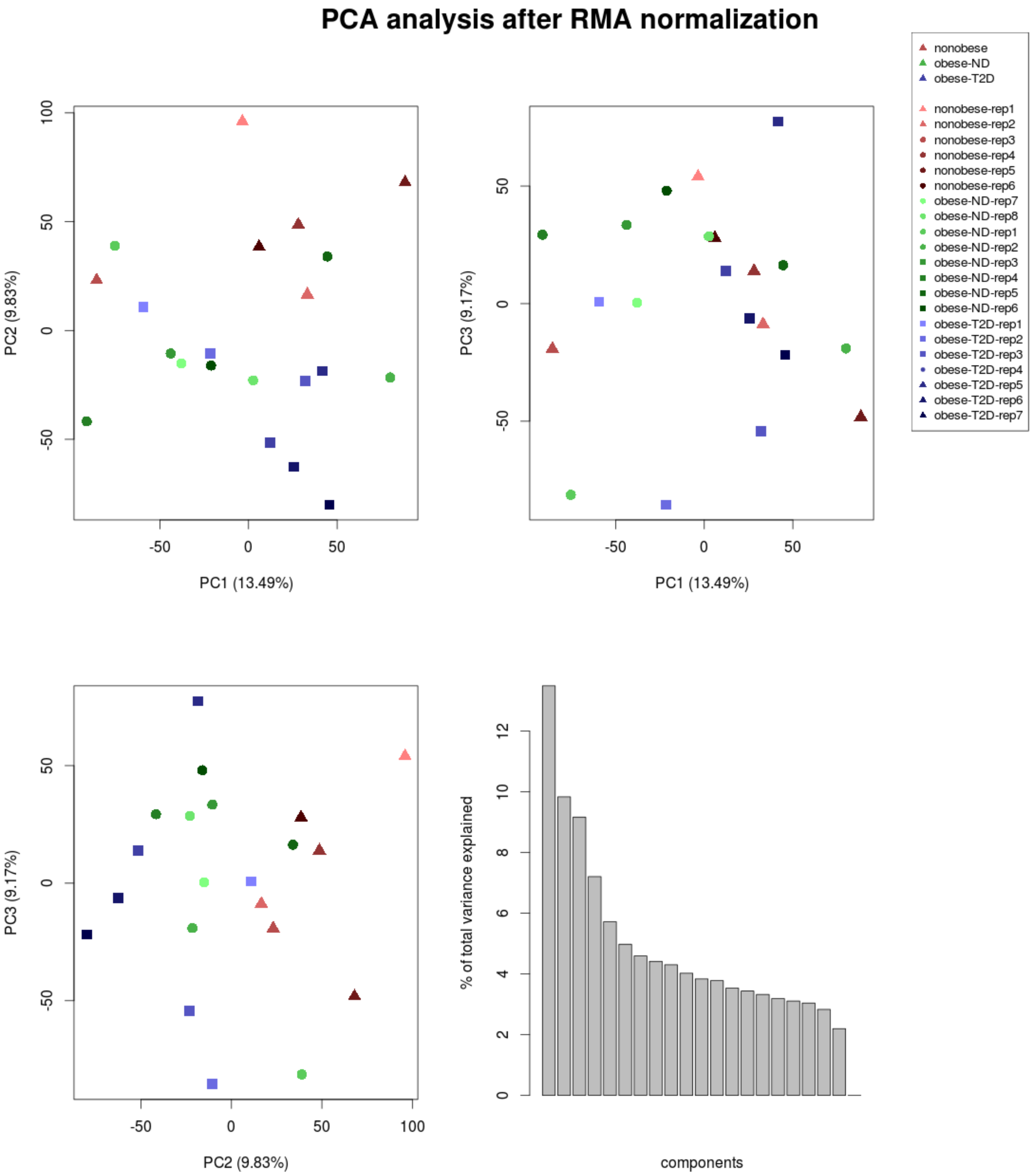
PCA analysis after RMA normalization
PC1 (13.49%)
PC2 (9.83%)
PC3 (9.17%)
components
% of total variance explained
nonobese
obese-ND
obese-T2D
nonobese-rep1
nonobese-rep2
nonobese-rep3
nonobese-rep4
nonobese-rep5
nonobese-rep6
obese-ND-rep7
obese-ND-rep8
obese-ND-rep1
obese-ND-rep2
obese-ND-rep3
obese-ND-rep4
obese-ND-rep5
obese-ND-rep6
obese-T2D-rep1
obese-T2D-rep2
obese-T2D-rep3
obese-T2D-rep4
obese-T2D-rep5
obese-T2D-rep6
obese-T2D-rep7

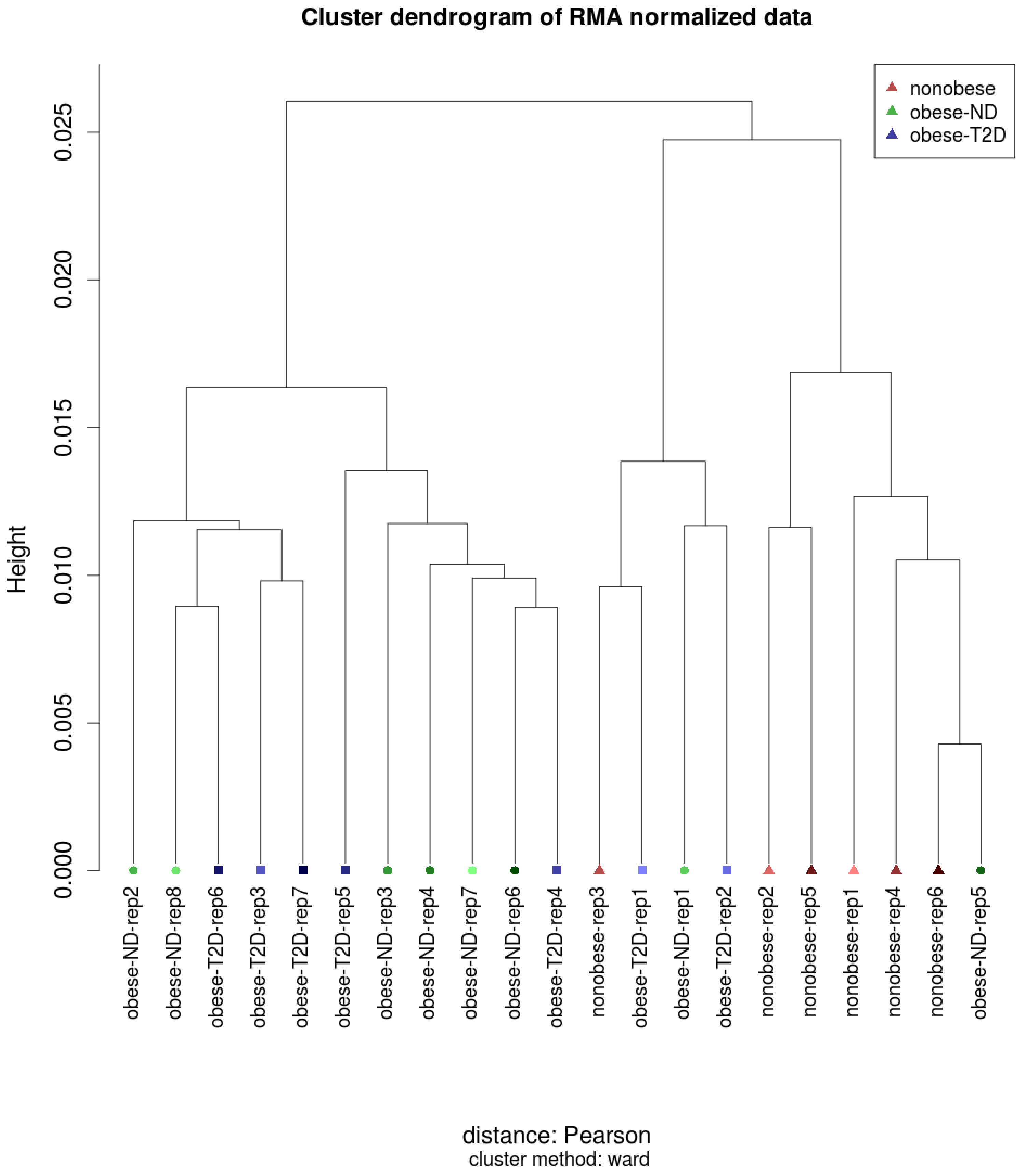
Cluster dendrogram of RMA normalized data
nonobese
obese-ND
obese-T2D
Height
0.000
0.005
0.010
0.015
0.020
0.025
obese-ND-rep2
obese-ND-rep8
obese-T2D-rep6
obese-T2D-rep3
obese-T2D-rep7
obese-T2D-rep5
obese-ND-rep3
obese-ND-rep4
obese-ND-rep7
obese-ND-rep6
obese-T2D-rep4
nonobese-rep3
obese-T2D-rep1
obese-ND-rep1
obese-T2D-rep2
nonobese-rep2
nonobese-rep5
nonobese-rep1
nonobese-rep4
nonobese-rep6
obese-ND-rep5
distance: Pearson
cluster method: ward
