# Supplementary material for: Biological Pathways Leading From ANGPTL8 to Diabetes Mellitus–A Co-expression Network Based Analysis
Source: Front Physiol. 2018 Dec 21;9:1841. doi: 10.3389/fphys.2018.01841 (PMC6309236; doi:10.3389/fphys.2018.01841)
Supplement: Data Sheet 3 — The complete R Code used to perform WGCNA. [file Data_Sheet_3.PDF]

```
#########################################################################################
###############################

# Data input and cleaning

#########################################################################################
###############################

library(WGCNA)

options(stringsAsFactors = FALSE);


dbData = read.table("NormData_GSE64998.txt", as.is=T,sep="\t",header=T);

test=dbData

dbData=as.data.frame(dbData[, -c(1)]);

rownames(dbData)=test$ENTREZG_ID

dbData<-dbData[rowMeans(dbData)>5,]

dbExpr<-as.data.frame(t(dbData))


# To check for genes and samples with too many missing values:

gsg = goodSamplesGenes(dbExpr, verbose = 3);

gsg$allOK


# If the last statement returns TRUE, all genes have passed the cuts. If not, we remove the offending
genes and samples

# from the data.


# Next we cluster the samples (in contrast to clustering genes that will come later) to see if there are any
obvious

# outliers

sampleTree = hclust(dist(dbExpr), method = "ward.D");
```

```
# Plot the sample tree: Open a graphic output window of size 12 by 9 inches
# The user should change the dimensions if the window is too large or too small.
sizeGrWindow(12,9)
sizeGrWindow(14,8)
par(cex = 0.6);
par(mar = c(0,4,2,0))
plot(sampleTree, main = "Sample clustering to detect outliers", sub="", xlab="", cex.lab = 1.5,
    cex.axis = 1.5, cex.main = 2)

###############################################################################################
#####################################
#  Automatic network construction and module detection
###############################################################################################
#####################################

# First step is Choosing the soft-thresholding power: analysis of network topology

# Choose a set of soft-thresholding powers
powers = c(c(1:15), seq(from = 16, to=20, by=2))
# Call the network topology analysis function
sft = pickSoftThreshold(dbExpr, powerVector = powers, verbose = 5)
# Plot the results:
sizeGrWindow(9, 5)
par(mfrow = c(1,2));
cex1 = 0.9;
# Scale-free topology fit index as a function of the soft-thresholding power
plot(sft$fitIndices[,1], -sign(sft$fitIndices[,3])*sft$fitIndices[,2],
    xlab="Soft Threshold (power)",ylab="Scale Free Topology Model Fit,signed R^2",type="n",
    main = paste("Scale independence"));
```

```
text(sft$fitIndices[,1], -sign(sft$fitIndices[,3])*sft$fitIndices[,2],
    labels=powers,cex=cex1,col="red");
# this line corresponds to using an R^2 cut-off of h
abline(h=0.90,col="red")
# Mean connectivity as a function of the soft-thresholding power
plot(sft$fitIndices[,1], sft$fitIndices[,5],
    xlab="Soft Threshold (power)",ylab="Mean Connectivity", type="n",
    main = paste("Mean connectivity"))
text(sft$fitIndices[,1], sft$fitIndices[,5], labels=powers, cex=cex1,col="red")

# Second step is the one-step network construction and module detection
#64998>5
net = blockwiseModules(dbExpr, power = 14,
                TOMType = "unsigned", minModuleSize = 30,
                reassignThreshold = 0, mergeCutHeight = 0.25,
                numericLabels = TRUE, pamRespectsDendro = FALSE,
                saveTOMs = TRUE,
                saveTOMFileBase = "dbTOM",
                verbose = 3)
table(net$colors)

# Creatig a hierarchical clustering dendrogram (tree) for the module identification
# open a graphics window
sizeGrWindow(12, 9)
# Convert labels to colors for plotting
mergedColors = labels2colors(net$colors)
# Plot the dendrogram and the module colors underneath
plotDendroAndColors(net$dendrograms[[1]], mergedColors[net$blockGenes[[1]]],
               "Module colors",
```

```
                  dendroLabels = FALSE, hang = 0.03,
                  addGuide = TRUE, guideHang = 0.05)

# We now save the module assignment and module eigengene information necessary for subsequent analysis
moduleLabels = net$colors
moduleColors = labels2colors(net$colors)
MEs = net$MEs;
geneTree = net$dendrograms[[1]];
save(MEs, moduleLabels, moduleColors, geneTree,
  file = "gse64998-networkConstruction-auto.RData")
#The end
```
